# A Novel In Situ Sol-Gel Synthesis Method for PDMS Composites Reinforced with Silica Nanoparticles

**DOI:** 10.3390/polym16081125

**Published:** 2024-04-17

**Authors:** Aldo Cordoba, Juan Valerio Cauich-Rodríguez, Rossana Faride Vargas-Coronado, Rodrigo Velázquez-Castillo, Karen Esquivel

**Affiliations:** 1 Graduate and Research Division, Engineering Faculty, Universidad Autónoma de Querétaro, Cerro de las Campanas, Queretaro 76010, Mexico; acordoba07@alumnos.uaq.mx (A.C.); rodrigo.velazquez@uaq.mx (R.V.-C.); 2 Centro de Investigación Científica de Yucatán, Unidad de Materiales, C. 43 No. 130 x 32 y 34, Col. Chuburná de Hidalgo, Merida 97205, Mexico; jvcr@cicy.mx (J.V.C.-R.); ross@cicy.mx (R.F.V.-C.)

**Keywords:** in situ synthesis, nanocomposites, sol-gel method, polymer coatings, reinforcers

## Abstract

The addition of nanostructures to polymeric materials allows for a direct interaction between polymeric chains and nanometric structures, resulting in a synergistic process through the physical (electrostatic forces) and chemical properties (bond formation) of constituents for the modification of their properties and potential cutting-edge materials. This study explores a novel in situ synthesis method for PDMS-%SiO_2_ nanoparticle composites with varying crosslinking degrees (PDMS:TEOS of 15:1, 10:1, and 5:1); particle concentrations (5%, 10%, and 15%); and sol-gel catalysts (acidic and alkaline). This investigation delves into the distinct physical and chemical properties of silicon nanoparticles synthesized under acidic (SiO_2_-a) and alkaline (SiO_2_-b) conditions. A characterization through Raman, FT-IR, and XPS analyses confirms particle size and agglomeration differences between both the SiO_2_-a and SiO_2_-b particles. Similar chemical environments, with TEOS and ethanol by-products, were detected for both systems. The results on polymer composites elucidate the successful incorporation of SiO_2_ nanoparticles into the PDMS matrix without altering the PDMS’s chemical structure. However, the presence of nanoparticles did affect the relative intensities of specific vibrational modes over composites from −35% to 24% (Raman) and from −14% to 59% (FT-IR). The XPS results validate the presence of Si, O, and C in all composites, with significant variations in atomic proportions (C/Si and O/Si) and Si and C component analyses through deconvolution techniques. This study demonstrates the successful in situ synthesis of PDMS-SiO_2_ composites with tunable properties by controlling the sol-gel and crosslinking synthesis parameters. The findings provide valuable insights into the in situ synthesis methods of polymeric composite materials and their potential integration with polymer nanocomposite processing techniques.

## 1. Introduction

Silicon polymers are widely used in various applications, owing to their being lightweight and flexible and their ease of sterilization, low cost, straightforward manufacturing processes, and remarkable resistance to physical aging and degradation. This extensive array of properties has enabled their application across virtually all industrial and research sectors [1]. Aligned with these attributes, the incorporation of nanoscale materials into polymeric materials has been the subject of extensive exploration in recent decades [2]. The incorporation of nanomaterials of diverse chemical (metals, ceramics, and polymers) and physical nature (nanoparticles, nanowires, nanoplates, etc.) has constituted a field of research in composite materials, being the nanocomposite polymers formed by the integration of nanostructured materials with a polymeric material as the matrix [3]. This investigation pursues a response to the continuous demand for materials with enhanced performance that the evolving industries require [4].

In recent years, numerous research efforts have focused on the design and evaluation of polymeric composites based on silicon polymers with nanostructures of varying morphologies and chemical nature [5]. This widespread investigation has been demonstrated in research that aimed to improve the physiochemical properties of polymeric materials by incorporating reinforcing nanostructures such as carbon nanotubes [6], metallic nanoparticles on polymeric fibers [7], and metal oxides [8] with an emphasis on applications in electronic and optical circuits. Other approaches seek to broaden the versatility of these polymeric matrices by imparting anticorrosive [9] or antimicrobial properties [10]. Similarly, the mechanical properties of different polymerics, including tensile strength, elastic modulus, and hardness [11,12], as well as barrier properties and separation membrane characteristics [13] have been modified through the use of nanostructured metal oxides.

Conventionally, two pathways are followed for obtaining these nanocomposite systems, an ex situ and in situ synthesis, each with thoroughly studied advantages and disadvantages [3]. In contrast to the ex situ method, the in situ composite synthesis has proven to be a potentially effective approach in addressing common challenges associated with the use of nanostructures, such as extensive synthesis times and limited polymer–reinforcement interactions often caused by agglomeration [14]. Among the most widely used synthesis methods, the sol-gel method stands out as the most extensively studied technique due to the easy integration of two synthesis methods and conditions for both materials [15].

The in situ formation of nanocomposite systems using substrates such as polydimethylsiloxane (PDMS) and metal oxide nanoparticles has presented significant advantages for the development of superhydrophobic surfaces [16], antimicrobial properties [17], fouling prevention [8], and insulating coatings [18]. The use of silica nanoparticles (SiO_2_) is particularly noteworthy in this context, where modifications in optical, mechanical, electrical, and chemical properties are achieved based on their size, surface chemistry, and porosity degree, which are adaptable during synthesis and under certain conditions [19].

Despite the advantages presented by in situ methods, a competition between polymerization reactions and particle synthesis, along with the presence of by-products formed in the chemical environment, sometimes proves incompatible, diminishing the formation of nanocomposite systems through this method [20].

In recent years, significant progress has been made in the development of in situ nanocomposites by utilizing polymeric materials as growth matrices for nanostructures, leading to the emergence of metal–polymer [21], polymer–polymer [22], and ceramic–polymer composite systems [23]. However, these advancements are hindered by the incomplete interaction between the polymer and nanostructures, resulting in surface composite materials rather than achieving full polymer–nanostructure integration. Despite the formation of composite systems, this superficial interaction restricts the final material properties to the surface level, failing to extend throughout the entire polymer matrix [24]. Furthermore, in situ nanocomposites such as Ag-Collagen [25] and ZnO-Chitosan [23] have been obtained through thermal processes to promote particle synthesis reactions and polymerization processes. However, this approach presents challenges in the composite fabrication methods, often requiring phase separation processes [21,22].

In response to these challenges, the versatility offered by the sol-gel method to modify the final properties of the material through synthesis conditions may present a potential solution for the development of in situ nanocomposites. By applying different synthesis conditions, a significant influence on the reactions occurring in polymer composite systems, such as hydrolysis, condensation, and crosslinking, has been observed [22]. 

Among these synthesis conditions, the hydrogen potential (pH) has proven to significantly alter the physicochemical properties of nanoparticles, as well as the hydrolysis and condensation reaction rates [15].

Therefore, the development of a methodology that mitigates the competition of chemical reactions during synthesis and facilitates integration between the polymeric and nanoparticulate systems becomes an essential tool for overseeing the chemical processes occurring during nanocomposite manufacturing. 

This research proposes a novel methodology for obtaining a polymeric nanocomposite with a PDMS matrix through the in situ synthesis of SiO_2_ nanoparticles via the sol-gel method, utilizing pH as a control variable for initiating hydrolysis–condensation reactions and crosslinking processes with acidic and alkaline environments. This proposal aims to demonstrate a pathway that leverages the benefits of in situ synthesis to enhance the interaction and dispersion of nanostructured systems, as well as to reduce the competition of chemical reactions during the synthesis process.

## 2. Materials and Methods

Before the experimental procedures, all glassware utilized in the synthesis was thoroughly washed with ethanol, rinsed with distilled water, and dried with absorbent paper to eliminate potential impurities. The used reagents for the experimental procedures were dimethyl ketone (C_3_H_6_O) (99.5%, Meyer^®^, Mexico City, Mexico); hydroxy-terminated polydimethylsiloxane (PDMS-OH) (2550–3570 cSt, Sigma Aldrich^®^, Milwaukee, WI, USA); tetraethylorthosilicate (TEOS) (98% Sigma Aldrich^®^, Wuxi, China); ammonium hydroxide (NH_4_OH) (28% J.T.Baker^®^, Madrid, Spain); nitric acid (HNO_3_) (65–70% J.T.Baker^®^, Madrid, Spain); and dibutyltin dilaurate (DBTDL) (95% Sigma Aldrich^®^, Milwaukee, WI, USA).

### 2.1. SiO_2_ Sol-Gel Synthesis

To ascertain the physicochemical characteristics of the components that constituted the polymeric composites, the synthesis of SiO_2_ nanoparticles was conducted by employing the Stöber method [26,27] and a previously developed methodology in our research group [28,29] for both acidic and alkaline media. 

Inside an inert environment (N_2_), acetone was stirred for 15 min. TEOS was added dropwise to the solution and mixed for 20 min. A weight ratio of 1:1.42 (C_3_H_6_O:TEOS) was used. For the alkaline SiO_2_ synthesis, the hydrolysis reaction was initiated by adding ammonium hydroxide (NH_4_OH) dropwise as a catalyst until the pH reached 10 and by stirring for 2 h. The obtained gel was poured into a plastic petri dish and dried at room temperature for 24 h. For the synthesis under acidic conditions, a nitric acid (HNO_3_) catalyst was added dropwise until the pH reached 2. The stir and dry conditions for the acid synthesis were the same as those of the alkaline synthesis.

The synthesized particles were subjected to various characterization techniques. For morphological observation, TEM imaging was obtained using a JEM 2000FX transmission electron microscope, operating at 15 kV (JEOL USA; Peabody, MA, USA). XRD analyses were performed through a D8 Advance diffractometer (Bruker; Karlsruhe, Baden, Germany) with a Cu anode (20 kV, 20 mA, and λ = 1.5406 Å). The scanned angular range was from 10 to 80 degrees in 2θ with a rate of 10° per minute. Raman spectroscopy was performed using a D2700M inVia Raman Microscope (Renishaw; Wetzlar, Hesse, Germany) equipped with an NdYHA laser with a wavelength of λ = 523 nm. FT-IR analyses were performed with a Nicolet 8700 FT-IR spectrometer (Thermo Scientific; Madison, WI, USA) equipped with ATR equipment and operated under Absorbance mode. The XPS analysis was recorded using a K-Alpha Surface Analyzer (Thermo Scientific; Madison, WI, USA) equipped with a multichannel detector with X-ray 400 μm-FG ON (400 μm). 

### 2.2. Synthesis of PDMS/NP-SiO_2_ Nanocomposites

The in situ synthesis of PDMS-%SiO_2_ nanocomposites was carried out utilizing the sol-gel method and the crosslinking reaction of the hydroxyl-terminated PDMS matrix using magnetic and ultrasonic stirring techniques, as detailed in prior publications [28,29]. The synthesis initiates with a solution of acetone and the PDMS with a 1:0.6 weight ratio (PDMS: C_3_H_6_O). Mechanical stirring was applied to homogenize the solution for 1 h. TEOS was added according to the desired nanoparticle concentration (0%, 5%, 10%, and 15% by weight). The reported particle concentration values were derived from the weight particle yields obtained during the SiO_2_ sol-gel synthesis process and previous research [29]. Through this in situ synthesis, the concentration represented a categorical variable that denotes a relative number of particles incorporated within the composite.

To initiate the hydrolysis process and the formation of SiO_2_ nanoparticles, the corresponding alkaline or acid catalyst (NH_4_OH or HNO_3_) was added dropwise until a pH of 10 (alkaline) or 2 (acidic) was achieved [27]. The solution was stirred for 2 h and then neutralized (pH 7). TEOS was added to the solution based on the desired matrix:crosslinker weight ratio (5:1, 10:1, and 15:1), and the mixture was stirred for 1 h. The solution was degassed by using an ultrasonic bath for 15 min to eliminate the remaining air bubbles. The solution was mechanically stirred for 5 min to reach room temperature.

The crosslinking reaction was initiated by adding the DBTDL catalyst dropwise with a weight ratio of 1:0.02 (PDMS:DBTDL). Finally, the solution was mechanically stirred for 5 min, deposited on an acrylic substrate, and left to cure at room temperature for 24 h.

Raman, FT-IR spectroscopy, and XPS analyses were conducted to identify chemical species with the same configurations used for SiO_2_ NPs, such as characteristic vibrational modes. All of this was performed to determine the molecular composition of the synthesized composites.

## 3. Results

### 3.1. SiO_2_ NP Characterization

**Figure 1:**
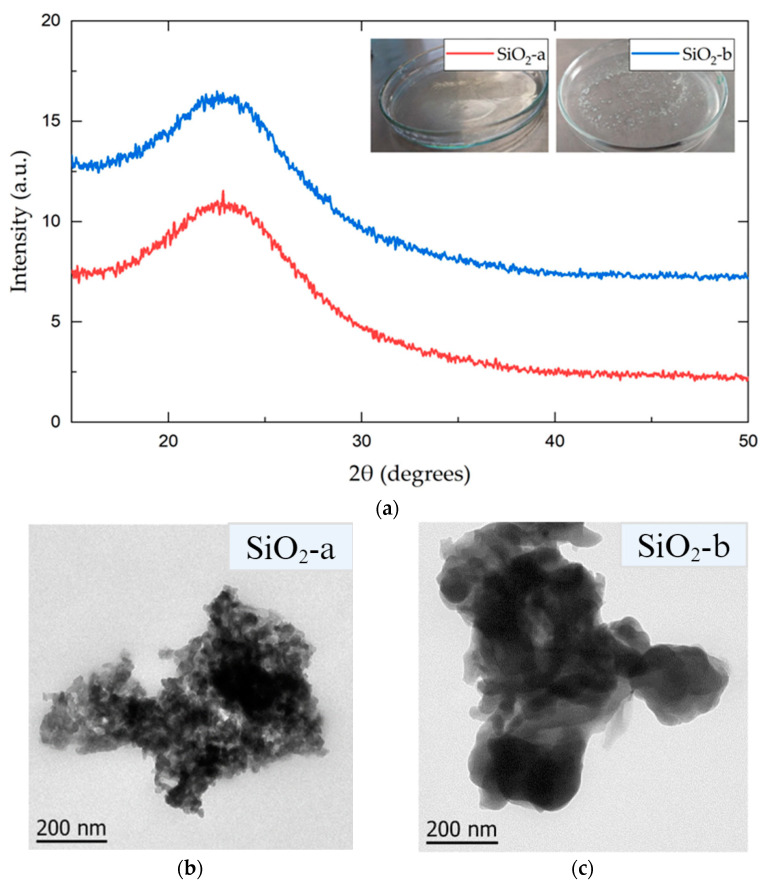
(**a**) X-ray diffractogram of sol-gel-synthesized SiO_2_ particles under acidic (SiO_2_-a) and alkaline (SiO_2_-b) conditions. TEM images of acidic (**b**) and alkaline (**c**) SiO_2_ nanoparticles.

The inset in Figure 1a depicts images of the obtained gels for both syntheses: acid (SiO_2_-a) and alkaline (SiO_2_-b) catalysis. Upon comparing both systems, a clear qualitative distinction was observed. In the alkaline gel, clusters of whitish particles were noticed, while in the acid system, a homogeneous and transparent solid gel was formed. The whitish hue in the alkaline catalysis is commonly observed in spherical SiO_2_ particles with particle sizes of around 100–150 nm [26]. In contrast, the transparent solution in the acidic catalysis could represent the formation of particles with dendritic structures with particles between 50 and 60 nm [27].

To determine the structural and morphological differences between the two synthesis processes, XRD and TEM techniques were performed from the synthesized SiO_2_ particles in both alkaline and acidic media. 

The diffractograms (see Figure 1a) show the typical broad signal generated by an amorphous material, corresponding to silica-based nanomaterials. This result is commonly observed in materials synthesized through the sol-gel process [29]. Both systems presented a wide Bragg reflection between 20° and 30°, with maximum intensities at 22.84° (SiO_2_-a) and 22.52° (SiO_2_-b). The TEM images of both the SiO_2_-a and SiO_2_-b particles (Figure 1b,c) show a high degree of agglomeration. However, there were significant differences in the particle size within the observed clusters. The SiO_2_-a particles, synthesized under acidic conditions, exhibited a smaller particle size and a lower agglomeration than those in SiO_2_-b, as previously reported [15].

**Figure 2:**
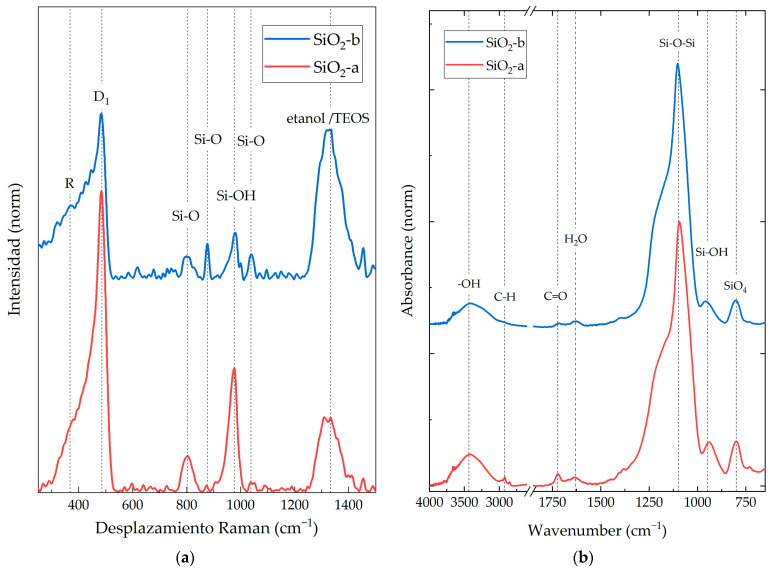
(**a**) Raman spectra and (**b**) FT-IR spectra of SiO_2_ nanoparticles synthesized by the sol-gel method under acidic (SiO_2_-a) and alkaline (SiO_2_-b) conditions.

Figure 2a shows the Raman spectra obtained for SiO_2_ particles. Six characteristic Raman-active vibrational modes were identified for both synthesis methods. R (380 cm^−1^) represents the flexion mode of oxygen atoms in Si-O-Si linkages, and D_1_ (484 cm^−1^) corresponds to the “breathing” relaxation mode of 4-member rings. The mode at 802 cm^−1^ is the vibration mode of the SiO_2_ network optical branch, while the 876 cm^−1^ is the contraction mode of Si-O-Si systems in the SiO_2_ network. The signal at 976 cm^−1^ corresponds to the vibration mode of the Si-OH molecule related to the surface silicon atoms. Finally, at 1041 cm^−1^, a stretching mode belonging to the Si-O-Si core bonds was observed [30]. The SiO_2_-a sample exhibited higher intensity in the six vibrational modes compared to the SiO_2_-b system. This may be attributed to a smaller particle size and a lower degree of agglomeration, as observed in the TEM images. This results in a larger surface area, which in turn leads to a stronger interaction with the Raman laser beam [31,32]. 

In addition to the six characteristic Raman-active vibrational modes, two additional peaks were observed at 1300 cm^−1^. These peaks belong to both TEOS and alcohol molecules, such as ethanol [33]. Ethanol is one of the main by-products of the sol-gel condensation reaction used to synthesize the SiO_2_ particles from TEOS [15]. The intensity of the alcohol-related peak was higher for the SiO_2_-b particles. This suggests that the SiO_2_-b particles contained a higher concentration of residual TEOS or ethanol.

In Figure 2b, the FT-IR spectrum of the synthesized SiO_2_ nanoparticles is presented. Both materials exhibited three characteristic vibrational modes of SiO_2_ structures within the analyzed wavelength range: 800 cm^−1^, corresponding to the asymmetric stretching vibration of Si-O-Si; 1093–1250 cm^−1^, representing the symmetric stretching vibration of cyclic and linear silica molecules; and 940–959 cm^−1^, corresponding to Si-OH stretching vibrations modes [34]. 

The FT-IR spectrum reveals a wide vibrational mode around 3700–3000 cm^−1^, which is attributed to the stretching vibration of -OH groups. These hydroxyl groups are found on the surface of nanoparticles synthesized by sol-gel methods [15], as well as their TEOS precursors and by-products like ethanol or water. Additionally, vibrational modes in the regions of 1620–1650 cm^−1^ and 1722–1750 cm^−1^ were observed. These were attributed to adsorbed water molecules [34] and the stretching vibrations of carbonyl groups (C=O) from acetone [35], respectively. For both registered peaks, SiO_2_-a presented an intense absorbance peak of this functional group. Vibrational mode 2950–2850 cm^−1^ corresponds to asymmetric stretching modes of C-H, which are characteristic of potential remnants of TEOS, ethanol, and acetone, as observed in Raman spectra [26].

**Figure 3:**
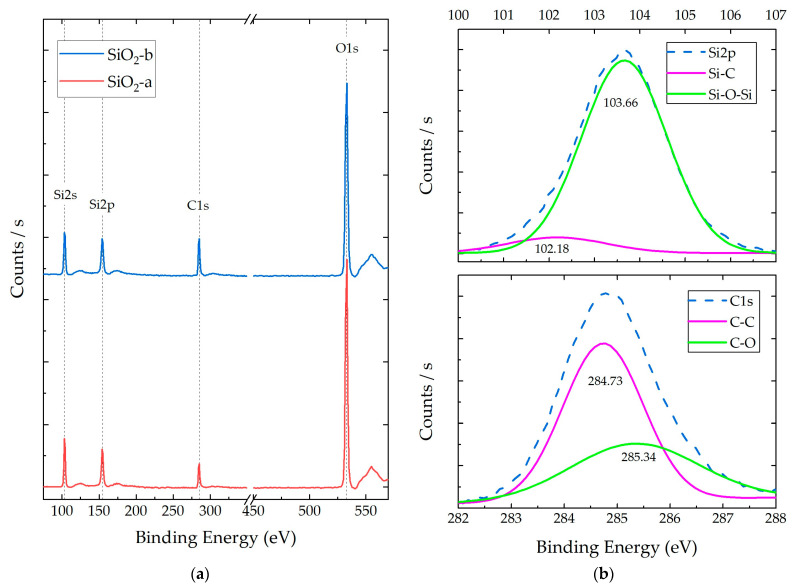
XPS spectra of synthesized SiO_2_ particles under acidic (SiO_2_-a) and alkaline (SiO_2_-b) conditions: (**a**) survey spectra and (**b**) representative deconvolution analysis for Si2p and C1 orbitals of SiO_2_-b.

Figure 3a shows the XPS survey spectra for both SiO_2_ particulate systems (a and b), and Figure 3b shows the corresponding scans and deconvolution for C1 and Si2p species for the SiO_2_-b particles (the SiO_2_-a deconvolution process can be consulted in Figure S1). This spectrum allowed for the identification of the chemical species (Si, C, and O) as well as their functional groups (deconvolution), intensities, atomic percentages, and relationships between species. The obtained XPS results and deconvolution processes are listed in Table 1. 

In the XPS spectra, the presence of atomic orbitals belonging to Si and O was determined, both associated with the silica (SiO_2_) structure, as reported in [36]. These systems presented Si:O atomic ratios of 1:1.54 (SiO_2_-a) and 1:1.44 (SiO_2_-b). Additionally, the presence of characteristic carbon orbitals (C1s) was observed, with a higher concentration in the SiO_2_-b samples. The evidence of these chemical species could correspond to precursors’ remnants (acetone and TEOS) and by-products of hydrolysis and condensation reactions (ethanol) [37].

The XPS deconvolution analysis of the SiO_2_ particles revealed the presence of two main peaks for the Si species, located at 101.57 and 103.52 eV for SiO_2_-a and at 102.18 and 103.66 for SiO_2_-b. These peaks can be attributed to the C-Si and Si-O-Si bonding configurations, respectively [38]. The C-Si bonds are present in the TEOS precursor, while the Si-O-Si bonds are characteristic of the SiO_2_ network [36]. For the C species, two main peaks were also observed at 284.66 and 285.37 eV for SiO_2_-a and at 284.73 and 285.34 eV for SiO_2_-b. These peaks could be assigned to the C-C and C-O bonding configurations, respectively [39]. These species are present in both the TEOS precursor and the ethanol by-product.

### 3.2. PDMS-%SiO_2_ Composite Chemical Characterization 

#### 3.2.1. Composite Raman Spectroscopy

**Figure 4:**
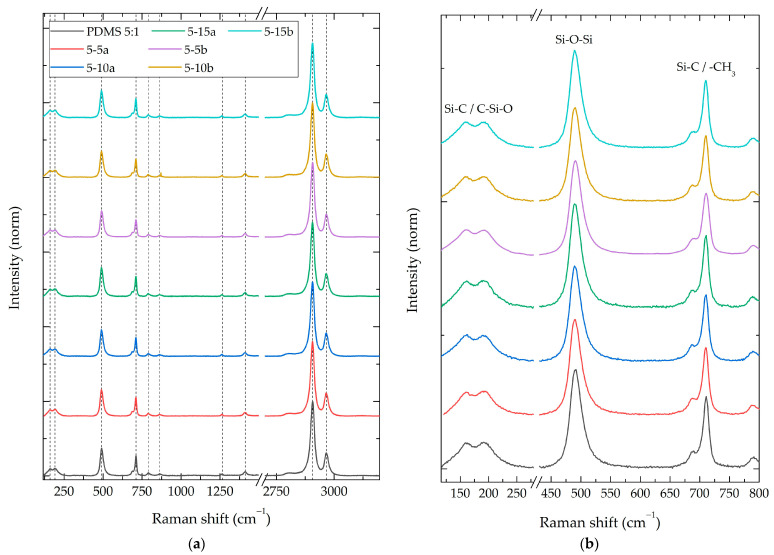
Raman spectra of PDMS 5:1 composite with different NPs SiO_2_ concentrations (5%, 10%, and 15%). (**a**) Survey spectra and (**b**) 145–800 cm^−1^ zoom in.

To identify the compositional differences between the synthesized composites, Raman, FT-IR, and XPS spectroscopies were performed. Figure 4a shows the Raman spectra of the PDMS 5:1-%SiO_2_ composite group. This group had a weight ratio of 5:1 (PDMS:TEOS) and normalized intensities. The remaining 14 spectra of all study groups can be consulted in Figure S2. Table 2 summarizes the Raman-active vibrational modes identified in all composite materials; the relative intensity of neat PDMS; and the changes in the intensity ratios of the composites with SiO_2_ nanoparticles (a and b) at concentrations of 5%, 10%, and 15%.

For all synthesized composites, Raman-active vibrational modes of the PDMS polymer were identified [40], without the presence of new peaks or vibration energy (Raman shift) modifications. A modification in the relative intensities of the vibrational modes was observed between the different composite groups, showing a relative intensity reduction of 35% and increments up to 24% compared to the PDMS without particles. 

The intensity results presented in Table 1 demonstrate a direct correlation between the weight ratio of PDMS:TEOS and the relative intensity of vibrational modes. The addition of SiO_2_ particles at lower concentrations leads to a reduction in the relative intensities of the observed vibrational modes. As the particle concentration rises, an increase in relative intensity was observed in the 150–800 cm^−1^ range (Figure 4b). This vibrational energy range aligns with the stronger characteristic Raman-active vibrational modes of the SiO_2_ nanoparticle systems (Figure 2).

Additionally, the vibrational mode at 2965 cm^−1^ also demonstrates a proportional increase in SiO_2_ concentration, suggesting the likely presence of residual TEOS/ethanol resulting from sol-gel reactions and crosslinking processes [26].

#### 3.2.2. Composite Fourier-Transform Infrared Spectroscopy (FT-IR)

**Figure 5:**
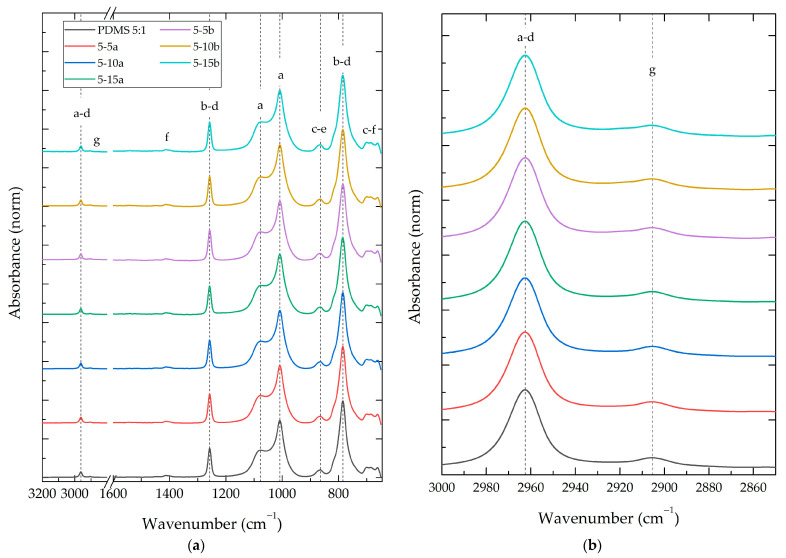
FT-IR spectra of PDMS 5:1 composite with different NP SiO_2_ types (**a**,**b**) and concentrations (5%, 10% and 15%). Letters a–g represent the observed vibration modes showed in Table 3.

Figure 5 shows the FT-IR spectrum of the PDMS 5:1-%SiO_2_ composite group. This group had a weight ratio of 5:1 (PDMS:TEOS) and normalized intensities. The remaining 14 FT-IR spectra of the study groups and PDMS, TEOS, and DBTDL reference spectra can be consulted in Figure S3. Table 3 displays the vibration wavelength of infrared-active characteristic bonds from crosslinked PDMS composites [41], along with their relative intensity and composites´ intensity shifts because of SiO_2_ nanoparticles (a and b) at theoretical weight concentrations of 5%, 10%, and 15%.

An FT-IR analysis of the PDMS-%SiO_2_ composites revealed minimal wavenumber shifts (<0.7%) in all identified vibrational modes compared to the neat PDMS:TEOS samples. This suggests a minimal influence of the SiO_2_ nanoparticles on the vibration energy of the polymer matrix, possibly due to a maintained similar chemical environment around the vibrating groups [10]. In contrast, the relative intensities of the vibrational modes in the composites exhibited significant changes compared to the neat PDMS:TEOS materials, ranging from −14% to +59% changes in relative intensities.

The relative intensity changes revealed a gradual increase in intensity in regions corresponding to vibrational modes characteristic of Si-O-Si bonds in SiO_2_ nanoparticles (1300–900 cm^−1^) [33]. This increase in intensity was more pronounced with higher SiO_2_ concentrations. Additionally, a significant increase in the intensity of vibrational modes was observed in the range of 3000 to 2900 cm^−1^. This increase could be attributed to an increase in the -OH groups present on the surface of the SiO_2_ particles [15].

#### 3.2.3. Composite X-ray Photoelectron Spectroscopy (XPS)

**Figure 6:**
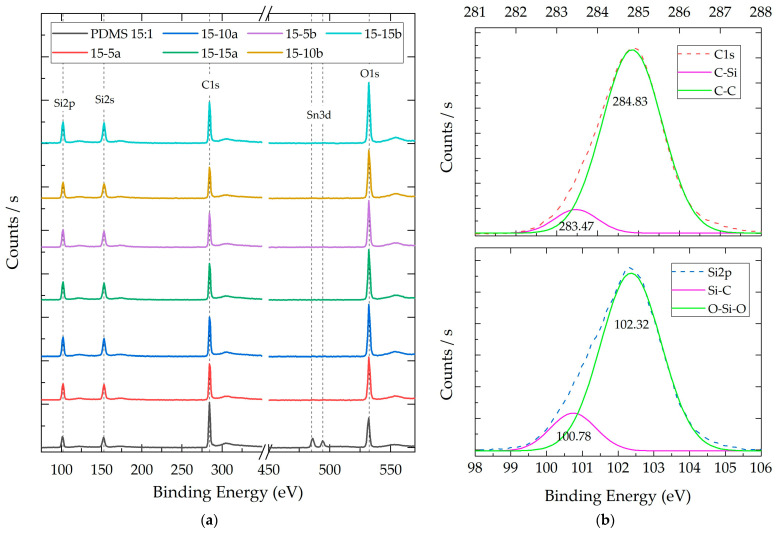
(**a**) Representative XPS survey spectra of composites with a 15:1 weight ratio (PDMS:TEOS) and different NP SiO_2_ concentrations (5%, 10%, and 15%). (**b**) Representative deconvolution analysis for Si2p and C1 orbitals in PDMS 15:1–10% SiO_2_-a.

Figure 6a shows the XPS results of the PDMS 15:1-%SiO_2_ composite group. PDMS 10:1 and PDMS 5:1 survey XPS spectra can be consulted in Figure S4. Figure 6b shows the representative deconvolution process performed on the Si and C species for each polymer composite for PDMS 15:1–10%SiO_2_-a. The deconvolution process of PDMS 15:1–10%SiO_2_-a and all other composites can be consulted in Figure S5 for C1s and in Figure S6 for S2p. This process identified the functional groups interacting with the Si and C species, as well as their concentration ratios.

Table 4 shows the results obtained from the XPS analysis. This table shows the identified species (Si, C, and O); the signal intensity; the atomic percentage; and the atomic ratio between the atomic species of the composite materials.

Through the application of deconvolution processes to the polymeric composites, the presence of two components related to the Si species was discerned. Both components were identified in all composites and presented binding energy averages of 100.68 ± 0.13 eV and 102.30 ± 0.08 eV. These contributions are attributed to the Si-C and Si-O-Si bonds, respectively [38]. The Si-C bonds are commonly associated with methyl (CH_3_) functional groups, serving as substituents intricately linked to the primary chain of PDMS [42]. Conversely, the Si-O-Si bonds can be associated with the main chain groups of the PDMS structure, in addition to being integral to the core of the SiO_2_ nanoparticles. 

The deconvolution results for the carbon atom allowed for the identification of two components in all composite materials. These components presented binding energy averages of 283.44 ± 0.13 eV and 284.81 ± 0.05 eV and can be attributed to the C-Si and C-C species, respectively [42]. Unlike the SiO_2_ nanoparticles, no components associated with C-O bonds, which typically manifest above 285 eV, were detected in these composites.

Through the atomic ratio comparison of the C/Si relation across the samples, it was observed that the PDMS:TEOS system with a lower degree of crosslinking (15:1) exhibits a higher carbon concentration with a 3.06 C/Si ratio. This ratio decreases for all other study groups, reaching a minimum of 1.74 in the PDMS5:1–10% b composite. Similarly, the atomic ratio of Si/O remains within the range of 0.82–0.84 for the polymers without SiO_2_ particles. The composites with SiO_2_ particles show an increase in the O/Si ratio, reaching up to 0.99. 

## 4. Discussion 

Physicochemical analyses of silicon nanoparticles synthesized under acidic (SiO_2_-a) and alkaline (SiO_2_-b) conditions revealed distinct physical and chemical properties. Although both exhibited amorphous structures, as confirmed by XRD diffractograms (Figure 1a), macroscopic differences during gel-formation behaviors were observed. This suggests that the nanoparticles undergo distinct reaction pathways during the sol-gel process, which is influenced by the medium’s pH, as previously reported [43]. 

As the sol-gel reactions advance, the functional groups bonded to the TEOS orthosilicate transform from ≡Si-O-Et to ≡Si-OH and ≡Si-O-Si≡, presenting a progressive decrease in electron density. In acidic media, H^+^ ions preferentially react with species of a higher electron density (≡Si-O-Et), promoting hydrolysis and particle formation. Conversely, in an alkaline medium, OH^-^ ions preferentially react with molecules of a lower electron density (≡Si-O-Si≡ or ≡Si-OH), favoring condensation and particle growth [15].

This expected behavior is consistent with the morphologies observed in the TEM images (Figure 1b,c). The SiO_2_-a particles exhibited smaller sizes and less agglomeration compared to the SiO_2_-b particles, which can be explained by the different reaction routes. In SiO_2_-a, promoting hydrolysis reactions prioritizes the formation of new particles with smaller sizes. In contrast, SiO_2_-b prioritizes particle growth due to the promotion of condensation, resulting in larger and more condensed particles [43].

The Raman, FT-IR, and XPS analyses confirmed that both the SiO_2_-a and SiO_2_-b particles possess similar chemical environments, containing the same chemical species, exhibiting similar vibrational energies, and displaying consistent O/Si atomic ratios (1.45 and 1.54, respectively). These results align with chemical properties encountered in silica sol-gel particles [30,33,36]. The presence of TEOS and ethanol molecules, common by-products in sol-gel processes, was confirmed by the identification of C-Si, C-O, C-C, and C-H species (Figure 6). A higher concentration of these species was observed in the SiO_2_-b particles, as evidenced by the stronger relative intensities in Raman and FT-IR spectra and a higher C1 atomic ratio in the XPS analysis (Table 4). Conversely, the SiO_2_-a particles presented a higher concentration of Si-OH species, which have been attributed to a greater hydroxylated surface area provoked by a smaller particle size for sol-gel particles without thermal treatments [15]. These results agree with the morphological differences between both particle systems.

Variations in species concentration between the two particle types are crucial as they will coexist with PDMS during in situ synthesis. Unlike previous studies where sol-gel by-products were eliminated through thermal treatments or filtration for chemical characterization [29], this analysis required the identification of all present species to identify those that will not be removed during the in situ process.

The Raman and FT-IR spectroscopy results of PDMS-%SiO_2_ composites revealed no new vibrational modes and only minor modifications (less than 1%) in the wavenumber or Raman shift of the PDMS’s bonds. This confirmed that the crosslinking density reached in these materials and the presence of particles did not alter the vibrational energy of the crosslinked PDMS, similar to observations for ex situ-synthesized PDMS-SiO_2_ composites [29]. This behavior can be attributed to the structural similarities between the nanoparticles and PDMS (Si-O and Si-OH bonds), with TEOS undergoing a chemical process analogous to nanoparticle formation during crosslinking [11].

While no new vibrational modes were observed, changes in the relative intensities of the composites’ spectra were identified compared to the PDMS:TEOS neat materials (15:1, 10:1, and 5:1). When the concentration of the crosslinking agent increased, the relative intensity of the PDMS vibrational modes remained constant or exhibited a slight increase. Conversely, in the presence of SiO_2_ particles, the composites displayed an initial decrease in the relative intensity of the vibrational modes in both the Raman and FT-IR spectra (−35% and −14%, respectively). This phenomenon has been previously reported and attributed to modifications in the polymer’s electron density because of the particle–polymer interaction [44]. The inherent competition between reactions during an in situ sol-gel synthesis can potentially alter the PDMS:TEOS ratio [3].

An increase in particle concentration within the composites resulted in a corresponding rise in the intensity ratio of specific spectral regions. In the Raman spectra, this enhancement was observed between 150 and 700 cm^−1^, while in the FT-IR spectra, it ranged from 900 to 1400 cm^−1^. A literature review and characterization results of SiO_2_ nanoparticles revealed that these spectral regions agree with the characteristic vibrational modes of SiO_2_. For the Raman spectra, the R and D_1_ vibrational modes (300–500 cm^−1^) corresponded to the “breathing” relaxation mode of Si-O rings [30]. Similarly, in the FT-IR spectra, the observed increase falls within the range of the asymmetric stretching mode of SiO_4_ core structures (1300–1000 cm^−1^) [33]. This has been related to the fact that an increase in the concentration of a particular species within a system could lead to corresponding alterations in the spectral regions associated with its characteristic vibrational modes, resulting in intensified or decreased peaks for the neat material [45].

The XPS analysis corroborated the presence of Si, O, and C in all the composites, aligning with the expected elements for silicon polymers [42]. Silicon and oxygen constitute the main chain of polysiloxane, while carbon corresponds to the methyl groups (-CH_3_) bonded to silicon atoms along the polymer chain. Trace amounts of tin (Sn^3d^) were also detected in the XPS spectra, exhibiting variations in intensity without a discernible pattern across the composites. The presence of these traces can be attributed to the DBTDL catalyst employed in the polymer crosslinking reaction [46].

While the same species were identified, their relative atomic proportions differed significantly between the composites. Notably, a reduction in the C/Si atomic ratio was observed with increasing SiO_2_ particle concentrations. This trend suggests that the decrease is not due to a lower abundance of carbon species but rather an increase in Si and O originating from the sol-gel particles. This hypothesis is further supported by the increasing C1 signal area in the composites compared to PDMS, which represents a more atomic concentration for the XPS technique [10].

Deconvolution revealed the absence of C-O species, suggesting a clear distinction from the synthesized SiO_2_ particles. This implies that no detectable remnants of precursors or by-products, such as TEOS or ethanol, are present on the XPS analysis surface [47]. These species might have been consumed during the polymer crosslinking reactions, as previous studies have demonstrated the feasibility of PDMS:crosslinker ratios up to 2:1 [48].

The O/Si ratio (0.82–0.99) displayed a slight increase with the particle concentration, yet it did not reach the values typically observed in SiO_2_ nanoparticles (1.54). This lines up with the O/Si ratios commonly reported for PDMS:TEOS structures [41]. This behavior can potentially be attributed to the presence of nanoparticles within the nanocomposite, as an increase in the concentration of SiO_2_ species likely originates an increase in Si and O species intensity [10].

The results indicate that composites with a higher particle concentration (PDMS-15%a and PDMS-15%b) exhibit a greater increase in the relative intensity of characteristic PDMS vibrational modes within the aforementioned spectral regions. Additionally, the composite PDMS-15%b displayed the most significant reduction in the Si/C ratio compared to all PDMS:TEOS investigated ratios (15:1, 10:1, and 5:1). These findings successfully confirm the presence and differentiation of SiO_2_ particles within the composite system compared to the response of PDMS with different crosslinker relations, as explored in previous studies [48].

## 5. Conclusions

This study investigated the in situ synthesis of PDMS-SiO_2_ nanoparticle composites, evaluating the influence of various parameters on their properties. These parameters included the crosslinking degree, particle concentration, and the type of sol-gel catalyst (acidic or alkaline).

Physicochemical analyses revealed distinct morphologies and surface chemistries for SiO_2_ nanoparticles prepared under acidic and alkaline conditions (SiO_2_-a and SiO_2_-b). These differences were attributed to the impact of pH on the reaction pathways during the sol-gel process. Notably, the analyses highlighted the importance of synthesis conditions in defining the final properties of the SiO_2_ particles, particularly regarding particle size, agglomeration, and the presence of by-products (TEOS and ethanol). This information provides valuable insights into how these factors might influence the interaction with PDMS during an in situ synthesis.

Raman, FT-IR, and XPS analyses confirmed the successful incorporation of SiO_2_ nanoparticles into the PDMS matrix without altering the PDMS’s chemical structure. However, the presence of nanoparticles did affect the relative intensities of specific vibrational modes, suggesting an interaction between the polymer and particles (variations from −35% to +24% for Raman and from −14% to +59% for FT-IR). Furthermore, the concentration of SiO_2_ particles significantly impacted the XPS results. Composites with higher particle concentrations (PDMS-15%SiO_2_-a and PDMS-15%SiO_2_-b) exhibited the maximum decrease in the C/Si ratio (minimum 1.715) and the higher increase in the intensity of SiO_2_ characteristic spectral regions (with a maximum O/Si ratio of 0.99), confirming the presence and modification response to different reticulation degrees and SiO_2_ concentrations.

These findings demonstrate the feasibility of the proposed in situ sol-gel method for synthesizing PDMS-SiO_2_ composites with tunable properties. By controlling synthesis parameters, researchers can tailor the final characteristics of the composites. This study lays the groundwork for the further exploration of the relationship between synthesis conditions and the resulting composite properties of this novel synthesis method.

Once the successful synthesis of these in situ nanocomposites is obtained, future research should focus on a more in-depth analysis of the reaction kinetics and the nature of the nanoparticle–polymer interaction. This deeper understanding will facilitate the optimization of properties like mechanical response, thermal resistance, hydrophobicity, etc. Ultimately, such advancements will bridge the gap between “one-step” synthesis methods and their integration into broader polymer production processes at both research and industrial scales.

## Figures and Tables

**Table 1:** XPS results of chemical species in synthesized SiO_2_ (acid and alkaline) nanoparticles and deconvolution process for S2p and C1 orbitals.

	Species	Binding Energy (eV)	Intensity (CPS)	FWHM (eV)	Area (CPS•eV)	Area (Norm)	Atomic Conc. (%)	Atomic Ratio *
SiO_2_-a	Si2p	101.57	1359.45	1.06	1507.74	0.027	31.82%	1
		103.52	28,287.24	1.86	54,919.59	0.973		
	C1s	284.66	14,530.61	1.48	22,874.40	0.619	19.21%	0.6
		285.37	4922.10	2.68	14,052.02	0.381		
	O1s	532.85	135,607.75	1.71	259,798.74	1	48.97%	1.54
SiO_2_-b	Si2p	102.18	1919.39	2.54	5097.19	0.086	31.15%	1
		103.66	23,610.95	2.21	54,497.88	0.914		
	C1s	284.73	18,567.63	1.78	35,174.67	0.635	23.98%	0.76
		285.34	6675.72	2.84	20,190.43	0.365		
	O1s	532.93	111,300.88	2.08	256,032.04	1	44.87%	1.44

* Atomic ratio between chemical species was calculated using the Si element as a calculus base.

**Table 2:** Raman-active vibrational modes for crosslinked PDMS-%SiO_2_ composites with Raman shift intensities varying by nanoparticle type and concentration [40].

PDMS 15:1									
	Vibrational Mode [30]	Raman Shift (cm^−1^)	0% (Norm)	a			b		
				5%	10%	15%	5%	10%	15%
1	Si-C torsion	157.40	0.075	1.66%	15.05%	22.79%	9.89%	11.56%	10.66%
2	C-Si-C deflection/C-Si-O deflection	192.63	0.078	5.22%	14.96%	24.07%	9.44%	11.54%	12.32%
3	Si-O-Si stretch	489.29	0.315	8.57%	12.73%	20.52%	10.85%	10.87%	10.18%
4	Si-C stretch	689.19	0.062	−6.23%	−0.55%	15.36%	−0.52%	−5.67%	−5.42%
5	Si–C symmetric stretch/CH_3_ rock	709.69	0.232	−2.45%	0.52%	11.44%	−0.35%	−0.87%	−1.28%
6	Si–C stretch/CH_3_ rock	788.66	0.038	−11.45%	−5.78%	16.30%	−8.77%	−14.81%	−7.58%
7	CH_3_ asymmetric stretch	861.97	0.029	−19.45%	−24.79%	−3.16%	−26.19%	−30.09%	−33.75%
8	Si-CH_3_ symmetric bending	1262.66	0.027	−18.41%	−18.05%	5.35%	−20.48%	−17.07%	−22.68%
9	CH_3_ asymmetric bending	1410.73	0.05	−11.46%	−8.39%	2.10%	−6.95%	−4.50%	−4.90%
10	CH_3_ symmetric stretch	2905.73	1	0.00%	0.00%	0.00%	0.00%	0.00%	0.00%
11	CH_3_ asymmetric stretch	2965.55	0.309	1.02%	−0.27%	1.29%	0.86%	0.70%	2.21%
**PDMS 10:1**									
	**Vibrational Mode [30]**	**Raman Shift** **(cm^−1^)**	**0%** **(Norm)**	**a**			**b**		
				**5%**	**10%**	**15%**	**5%**	**10%**	**15%**
1	Si-C torsion	158.70	0.089	10.49%	3.61%	6.68%	−7.15%	−5.83%	−4.21%
2	C-Si-C deflection/C-Si-O deflection	192.63	0.092	4.82%	7.44%	2.78%	−5.11%	−3.93%	−3.82%
3	Si-O-Si stretch	490.54	0.36	0.85%	1.74%	2.15%	−5.51%	−2.98%	−4.54%
4	Si-C stretch	687.98	0.063	2.67%	1.84%	2.82%	−7.86%	−6.05%	−6.14%
5	Si–C symmetric stretch/CH_3_ rock	710.89	0.252	0.12%	2.64%	−2.48%	−11.94%	−9.53%	−12.48%
6	Si–C stretch/CH_3_ rock	791.04	0.035	7.32%	18.21%	10.76%	−7.61%	−0.43%	2.20%
7	CH_3_ asymmetric stretch	864.32	0.025	0.81%	13.67%	3.35%	−8.93%	−9.13%	−4.39%
8	Si-CH_3_ symmetric bending	1262.66	0.027	−5.66%	4.56%	−21.92%	−15.65%	−24.98%	−15.94%
9	CH_3_ asymmetric bending	1412.88	0.05	−1.01%	5.61%	−5.93%	−5.47%	−7.43%	−3.61%
10	CH_3_ symmetric stretch	2906.33	1	0.00%	0.00%	0.00%	0.00%	0.00%	0.00%
11	CH_3_ asymmetric stretch	2965.55	0.311	−1.82%	−0.29%	−0.33%	−0.36%	0.49%	0.08%
**PDMS 5:1**									
	**Vibrational Mode [30]**	**Raman Shift** **(cm^−1^)**	**0%** **(Norm)**	**a**			**b**		
				**5%**	**10%**	**15%**	**5%**	**10%**	**15%**
1	Si-C torsion	160.01	0.097	−12.43%	4.69%	6.42%	−2.03%	−2.11%	1.69%
2	C-Si-C deflection/C-Si-O deflection	191.33	0.1	−10.55%	0.28%	4.83%	−6.90%	−5.89%	−0.85%
3	Si-O-Si stretch	491.78	0.373	−3.89%	−3.55%	4.92%	−5.31%	−5.55%	−1.36%
4	Si-C stretch	689.19	0.067	−4.59%	−4.90%	0.98%	−8.15%	−6.55%	−5.46%
5	Si–C symmetric stretch/CH_3_ rock	710.89	0.271	−6.82%	−7.87%	1.02%	−16.78%	−9.40%	−6.32%
6	Si–C stretch/CH_3_ rock	791.04	0.045	−18.12%	−13.81%	−0.22%	−24.81%	−18.59%	−16.84%
7	CH_3_ asymmetric stretch	861.97	0.03	−19.22%	−18.80%	0.49%	−34.65%	−23.14%	−15.60%
8	Si-CH_3_ symmetric bending	1265.97	0.03	−26.69%	−7.55%	−6.38%	−32.01%	−20.28%	−18.35%
9	CH_3_ asymmetric bending	1411.80	0.058	−16.67%	−16.06%	−3.87%	−23.19%	−15.18%	−16.39%
10	CH_3_ symmetric stretch	2907.15	1	0.00%	0.00%	0.00%	0.00%	0.00%	0.00%
11	CH_3_ asymmetric stretch	2966.36	0.311	−0.43%	0.67%	0.54%	−1.04%	0.80%	−0.06%

**Table 3:** FT-IR-active vibrational modes for crosslinked PDMS-%SiO_2_ composites and relative intensity variation [41].

PDMS 15:1									
	Wavenumber (cm^−1^)	Vibration Mode	0%	a			b		
				5%	10%	15%	5%	10%	15%
f	660.5	Si-CH_3_	0.14	−5.97%	−13.31%	−15.51%	−6.13%	−7.39%	−3.58%
c	684.61	Si-(CH_3_)_3_	0.14	−9.92%	−15.95%	−16.54%	−13.98%	−12.65%	−5.63%
c	699.55	Si-(CH_3_)_3_	0.14	−9.39%	−14.22%	−14.77%	−14.40%	−13.11%	−4.44%
b-d	784.4	Si(CH_3_)_2_-O-Si(CH_3_)_2_-/Si-(CH_3_)_3_	1	0.00%	0.00%	0.00%	0.00%	0.00%	0.00%
c-e	864.44	Si-(CH_3_)_3_/Si-OH	0.1	−2.91%	−9.36%	−10.64%	−8.84%	−8.16%	−2.86%
a	1008.11	Si-(CH_3_)n	0.76	2.33%	2.86%	4.52%	3.36%	5.09%	6.64%
a	1077.53	Si-(CH_3_)n	0.35	5.84%	6.04%	9.21%	8.91%	8.90%	14.60%
b-d	1257.36	Si(CH_3_)_2_-O-Si(CH_3_)_2_-/Si-(CH_3_)_3_	0.41	2.64%	1.42%	0.21%	5.77%	4.60%	0.03%
f	1412.12	Si-CH_3_	0.03	−11.68%	18.42%	−0.16%	−7.53%	−0.13%	−7.23%
g	2905.72	C-H	0.02	19.77%	19.94%	15.16%	21.14%	38.46%	26.67%
a,d	2962.61	SI(CH_3_)_n_/Si-(CH_3_)_3_	0.08	3.52%	2.91%	3.15%	7.21%	10.57%	6.78%
**PDMS 10:1**									
	**Wavenumber** **(cm^−1^)**	**Vibration Mode**	**0%**	**a**			**b**		
				**5%**	**10%**	**15%**	**5%**	**10%**	**15%**
f	660.5	Si-CH_3_	0.13	3.30%	−1.93%	−2.04%	14.20%	9.34%	0.18%
c	684.61	Si-(CH_3_)_3_	0.13	−3.72%	−5.46%	−2.15%	4.32%	−0.76%	−3.27%
c	699.55	Si-(CH_3_)_3_	0.13	−4.19%	−4.39%	−1.32%	2.21%	−1.33%	−3.40%
b-d	784.89	Si(CH_3_)_2_-O-Si(CH_3_)_2_-/Si-(CH_3_)_3_	1	0.00%	0.00%	0.00%	0.00%	0.00%	0.00%
c-e	864.44	Si-(CH_3_)_3_/Si-OH	0.1	−3.36%	−0.27%	−6.30%	3.40%	3.23%	−1.29%
a	1008.11	Si-(CH_3_)n	0.81	−8.03%	−4.26%	−2.26%	−3.50%	0.60%	0.62%
a	1077.05	Si-(CH_3_)n	0.37	−5.24%	−1.53%	−1.61%	5.56%	10.05%	10.24%
b-d	1257.36	Si(CH_3_)_2_-O-Si(CH_3_)_2_-/Si-(CH_3_)_3_	0.43	−9.49%	−6.03%	−5.15%	1.88%	1.91%	0.67%
f	1412.6	Si-CH_3_	0.03	−12.53%	4.92%	1.33%	34.26%	11.54%	25.17%
g	2905.72	C-H	0.02	−10.22%	−5.54%	−1.88%	26.84%	49.80%	19.59%
a,d	2962.125	SI(CH_3_)_n_/Si-(CH_3_)_3_	0.08	−9.16%	−5.96%	−3.00%	5.50%	12.44%	5.35%
**PDMS 5:1**									
	**Wavenumber** **(cm^−1^)**	**Vibration Mode**	**0%**	**a**			**b**		
				**5%**	**10%**	**15%**	**5%**	**10%**	**15%**
f	660.5	Si-CH_3_	0.13	−8.61%	−10.32%	−14.05%	−1.20%	−8.64%	−7.90%
c	685.09	Si-(CH_3_)_3_	0.13	−9.93%	−6.73%	−7.98%	−5.34%	−5.77%	−5.15%
c	699.55	Si-(CH_3_)_3_	0.13	−9.22%	−4.16%	−5.11%	−5.03%	−4.01%	−4.00%
b-d	784.4	Si(CH_3_)_2_-O-Si(CH_3_)_2_-/Si-(CH_3_)_3_	1	0.00%	0.00%	0.00%	0.00%	0.00%	0.00%
c-e	864.44	Si-(CH_3_)_3_/Si-OH	0.1	−6.99%	−3.89%	0.87%	−4.18%	−4.98%	−1.71%
a	1008.11	Si-(CH_3_)n	0.74	1.52%	2.59%	5.93%	4.57%	7.73%	8.11%
a	1077.05	Si-(CH_3_)n	0.35	1.38%	0.99%	7.03%	4.53%	7.50%	10.79%
b-d	1257.36	Si(CH_3_)_2_-O-Si(CH_3_)_2_-/Si-(CH_3_)_3_	0.4	0.16%	−1.37%	−1.94%	4.09%	2.58%	1.95%
f	1412.12	Si-CH_3_	0.03	−8.22%	1.21%	−9.22%	−13.18%	−10.33%	17.51%
g	2905.24	C-H	0.02	5.83%	8.11%	7.29%	59.76%	24.65%	17.05%
a,d	2962.61	SI(CH_3_)_n_/Si-(CH_3_)_3_	0.08	3.15%	2.38%	4.47%	14.40%	8.26%	5.90%

**Table 4:** XPS results of chemical species in PDMS-%SiO_2_ composites and deconvolution process for Si and C species.

PDMS 15:1									
Species		Binding Energy (eV)	Intensity (CPS)	FWHM (eV)	Area (CPS•eV)	Area (Norm)	Atomic Conc. (%)	Atomic Ratio *	Relation Shift
0%	Si2p	100.66	5009.84	1.54	8229.91	0.19	20.21%	1.000	-
		102.12	16,946.03	1.99	35,922.96	0.81			
	C1s	283.78	27,009.70	1.46	42,091.72	0.27	61.82%	3.059	-
		284.98	71,324.21	1.48	112,418.83	0.73			
	O1s	529.08	57,659.77	1.59	106,054.23	1.00	17.06%	0.844	-
	Sn3d	482.38	13,153.65	2.07	50,075.61	1.00	0.91%	0.045	-
5% a	Si2p	100.84	7870.52	1.60	13,418.41	0.22	25.54%	1.000	0.000
		102.30	25,513.39	1.80	48,861.13	0.78			
	C1s	283.39	10,737.10	1.21	13,828.33	0.09	53.62%	2.099	−0.959
		284.78	73,470.19	1.69	131,790.71	0.91			
	O1s	526.00	82,874.91	1.62	152,715.54	1.00	20.84%	0.816	−0.028
10% a	Si2p	100.78	7243.57	1.46	11,229.16	0.16	26.34%	1.000	0.000
		102.32	29,902.90	1.92	60,969.90	0.84			
	C1s	283.47	11,458.63	1.21	14,780.85	0.09	52.11%	1.978	−1.081
		284.83	78,640.31	1.71	143,052.16	0.91			
	O1s	526.00	94,731.26	1.63	175,305.35	1.00	21.55%	0.818	−0.026
15% a	Si2p	100.75	5996.81	1.49	9536.94	0.14	26.40%	1.000	0.000
		102.36	28,171.11	1.99	59,791.79	0.86			
	C1s	283.47	9441.53	1.23	12,359.82	0.08	51.30%	1.943	−1.116
		284.84	73,199.86	1.72	133,768.13	0.92			
	O1s	526.00	91,951.90	1.63	170,697.23	1.00	22.30%	0.845	0.001
5% b	Si2p	100.92	8986.55	1.54	14,696.63	0.22	27.37%	1.000	0.000
		102.40	26,703.67	1.81	51,393.55	0.78			
	C1s	283.45	11,868.31	1.36	17,127.24	0.13	50.46%	1.844	−1.215
		284.81	65,734.69	1.71	119,319.86	0.87			
	O1s	526.08	86,589.05	1.60	158,313.86	1.00	22.17%	0.810	−0.034
10% b	Si2p	100.54	3492.26	1.24	4616.74	0.08	26.08%	1.000	0.000
		102.31	24,503.38	2.17	56,610.91	0.92			
	C1s	283.48	10,530.95	1.17	13,088.25	0.10	50.40%	1.933	−1.126
		284.83	67,031.50	1.62	115,775.55	0.90			
	O1s	526.00	85,752.56	1.66	163,780.62	1.00	23.52%	0.902	0.058
15% b	Si2p	100.80	8372.29	1.53	13,591.69	0.17	27.19%	1.000	0.000
		102.38	33,761.94	1.88	67,485.20	0.83			
	C1s	283.48	15,322.67	1.20	19,562.28	0.12	49.84%	1.833	−1.226
		284.86	83,141.20	1.63	144,223.20	0.88			
	O1s	526.08	109,943.27	1.61	203,118.13	1.00	22.98%	0.845	0.001
**PDMS 10:1**									
**Species**		**Binding** **Energy (eV)**	**Intensity** **(CPS)**	**FWHM** **(eV)**	**Area** **(CPS•eV)**	**Area** **(Norm)**	**Atomic** **conc. (%)**	**Atomic** Ratio *	**Relation** **Shift**
0%	Si2p	100.39	233.10	1.33	329.17	0.07	25.95%	1.000	-
		102.25	2147.49	1.93	4408.81	0.93			
	C1s	283.15	438.10	1.41	657.33	0.06	52.68%	2.030	-
		284.79	5821.64	1.67	10,319.33	0.94			
	O1s	532.37	6454.43	1.52	11,236.48	0.82	21.37%	0.824	-
5% a	Si2p	100.68	5679.92	1.49	9038.24	0.15	25.62%	1.000	0.000
		102.30	25,206.02	1.84	49,283.79	0.85			
	C1s	283.35	9915.91	1.14	12,046.90	0.09	52.19%	2.037	0.007
		284.77	68,133.75	1.63	118,566.48	0.91			
	O1s	532.40	80,913.78	1.60	148,934.83	0.87	22.19%	0.866	0.043
10% a	Si2p	100.73	4663.48	1.43	7079.97	0.12	26.46%	1.000	0.000
		102.28	24,304.36	2.03	52,505.68	0.88			
	C1s	283.42	7865.35	1.19	9949.09	0.08	51.21%	1.935	−0.095
		284.77	62,767.35	1.75	116,978.52	0.92			
	O1s	532.42	82,356.19	1.57	148,456.83	0.84	22.33%	0.844	0.020
15% a	Si2p	100.83	7234.27	1.49	11,474.71	0.18	25.78%	1.000	0.000
		102.35	25,715.67	1.87	51,260.25	0.82			
	C1s	283.51	12,468.89	1.26	16,769.45	0.12	53.47%	2.074	0.044
		284.84	71,719.00	1.68	128,095.72	0.88			
	O1s	532.45	81,880.60	1.60	150,201.29	0.80	20.75%	0.805	−0.019
5% b	Si2p	100.61	5895.03	1.42	8900.28	0.15	25.07%	1.000	0.000
		102.26	25,818.22	1.89	51,972.14	0.85			
	C1s	283.37	12,287.41	1.18	15,383.71	0.11	53.09%	2.118	0.088
		284.76	74,468.33	1.60	126,702.54	0.89			
	O1s	532.36	85,026.62	1.63	158,707.76	0.87	21.83%	0.871	0.047
10% b	Si2p	100.59	4233.19	1.32	5938.89	0.09	26.89%	1.000	0.000
		102.35	27,144.65	2.00	57,705.79	0.91			
	C1s	283.35	9908.59	1.15	12,088.14	0.09	48.70%	1.811	−0.219
		284.77	67,028.12	1.62	115,583.89	0.91			
	O1s	532.41	94,238.89	1.60	173,925.70	0.91	24.41%	0.908	0.084
15% b	Si2p	100.51	2725.68	1.11	3232.76	0.05	27.17%	1.000	0.000
		102.43	26,596.30	2.20	62,273.85	0.95			
	C1s	283.44	8269.65	1.16	10,248.61	0.08	46.61%	1.715	−0.315
		284.84	64,416.59	1.66	113,486.00	0.92			
	O1s	532.54	99,166.24	1.67	188,714.81	0.97	26.22%	0.965	0.142
**PDMS 5:1**									
**Species**		**Binding** **Energy (eV)**	**Intensity** **(CPS)**	**FWHM** **(eV)**	**Area** **(CPS•eV)**	**Area** **(Norm)**	**Atomic** **conc. (%)**	**Atomic** Ratio *	**Relation** **Shift**
0%	Si2p	100.71	597.44	1.46	928.48	0.08	20.94%	1.000	-
		102.20	2736.10	2.07	6030.24	0.92			
	C1s	283.50	14,137.02	1.23	18,560.86	13.40	59.33%	2.833	-
		284.83	73,262.12	1.54	119,971.03	86.60			
	O1s	532.37	8445.93	1.66	16,195.07	0.83	17.30%	0.826	-
	Sn3d	486.17	5100.06	2.08	19,964.75	1.16	24.30%	1.160	-
5% a	Si2p	100.56	5524.30	1.42	8355.49	0.08	25.19%	1.000	0.000
		102.22	25,720.76	1.92	52,577.57	0.92			
	C1s	283.50	14,142.34	1.23	18,576.17	13.41	52.75%	2.094	−0.739
		284.83	73,258.51	1.54	119,956.16	86.59			
	O1s	532.41	84,872.40	1.62	158,280.22	0.88	22.06%	0.876	0.050
10% a	Si2p	100.57	5524.30	1.42	8355.49	0.08	25.99%	1.000	0.000
		102.22	25,720.76	1.92	52,577.57	0.92			
	C1s	283.50	10,205.15	1.26	13,657.94	10.70	50.60%	1.947	−0.886
		284.80	66,373.00	1.61	113,936.51	89.30			
	O1s	532.50	87,513.28	1.61	161,871.71	0.90	23.41%	0.901	0.075
15% a	Si2p	100.73	918.92	1.49	1456.09	0.07	25.58%	1.000	0.000
		102.23	4952.13	2.03	10,696.51	0.93			
	C1s	283.46	1798.26	1.36	2596.39	9.56	51.86%	2.027	−0.806
		284.81	13,236.78	1.74	24,556.04	90.44			
	O1s	532.52	16,964.46	1.59	31,657.35	0.88	22.55%	0.882	0.055
5% b	Si2p	100.59	2754.67	1.23	3605.89	0.04	24.15%	1.000	0.000
		102.36	19,783.53	1.97	41,467.13	0.96			
	C1s	283.18	3733.70	0.96	3806.93	3.66	51.91%	2.149	−0.684
		284.80	53,565.28	1.76	100,322.67	96.34			
	O1s	532.47	69,902.20	1.67	134,749.28	0.99	23.94%	0.991	0.165
10% b	Si2p	100.86	6752.94	1.47	10,592.09	0.09	27.22%	1.000	0.000
		102.37	26,917.55	1.95	55,842.61	0.91			
	C1s	283.51	11,301.88	1.22	14,633.87	10.87	50.57%	1.858	−0.976
		284.85	65,557.64	1.72	120,000.53	89.13			
	O1s	532.45	86,180.03	1.62	159,336.90	0.82	22.21%	0.816	−0.010
15% b	Si2p	100.64	4419.27	1.32	6206.52	0.06	27.44%	1.000	0.000
		102.35	25,854.94	2.01	553,93.02	0.94			
	C1s	283.48	9092.33	1.18	11,413.69	9.84	48.15%	1.755	−1.079
		284.79	59,787.62	1.64	104,542.07	90.16			
	O1s	532.45	88,917.78	1.60	163,418.89	0.89	24.41%	0.890	0.063

* Atomic ratio between chemical species was calculated using the Si element as a calculus base.

## Data Availability

Data are contained within the article.

## Supplementary Materials

The following supporting information can be downloaded at: https://www.mdpi.com/article/10.3390/polym16081125/s1. Figure S1: Deconvolution analysis for Si2p and C1 orbitals of SiO_2_-a particles. Figure S2: Raman spectra of (a) PDMS 15:1-%SiO_2_ and (b) PDMS 10:1-%SiO_2_ composites. Figure S3: FT-IR spectra of (a) PDMS 15:1-%SiO_2_ and (b) PDMS 10:1-%SiO_2_ composites. Figure S4: XPS spectra of (a) PDMS 10:1-%SiO_2_ and (b) PDMS 5:1-%SiO_2_ composites. Figure S5: Deconvolution analysis for C1 species of PDMS-%SiO_2_ composites: C1s (dash), C-Si (magenta), and C-C (green). Figure S6: Deconvolution analysis for Si2p species of PDMS-%SiO_2_ composites: Si2p (dash), Si-C (magenta), and O-Si-O (green).
