# Supplementary material for: A Novel In Situ Sol-Gel Synthesis Method for PDMS Composites Reinforced with Silica Nanoparticles
Source: Polymers (Basel). 2024 Apr 17;16(8):1125. doi: 10.3390/polym16081125 (PMC11053487; doi:10.3390/polym16081125)
Supplement: Supplementary file 1 [file polymers-16-01125-s001.zip › polymers-2954820-supplementary.pdf]

Suplementary Information

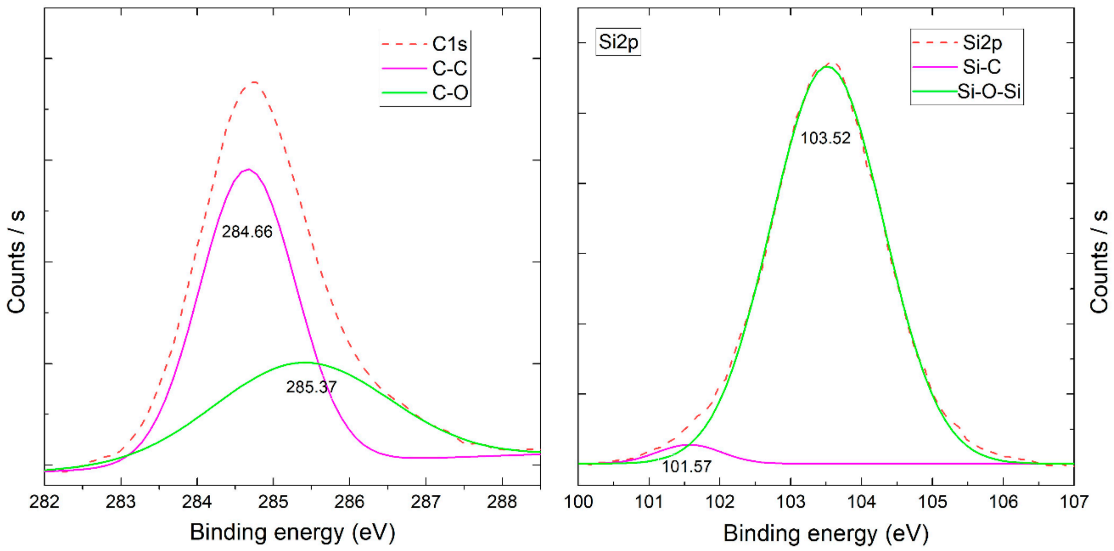

Figure S1. Deconvolution analysis for Si2p and C1s orbitals of SiO_2-a particles.

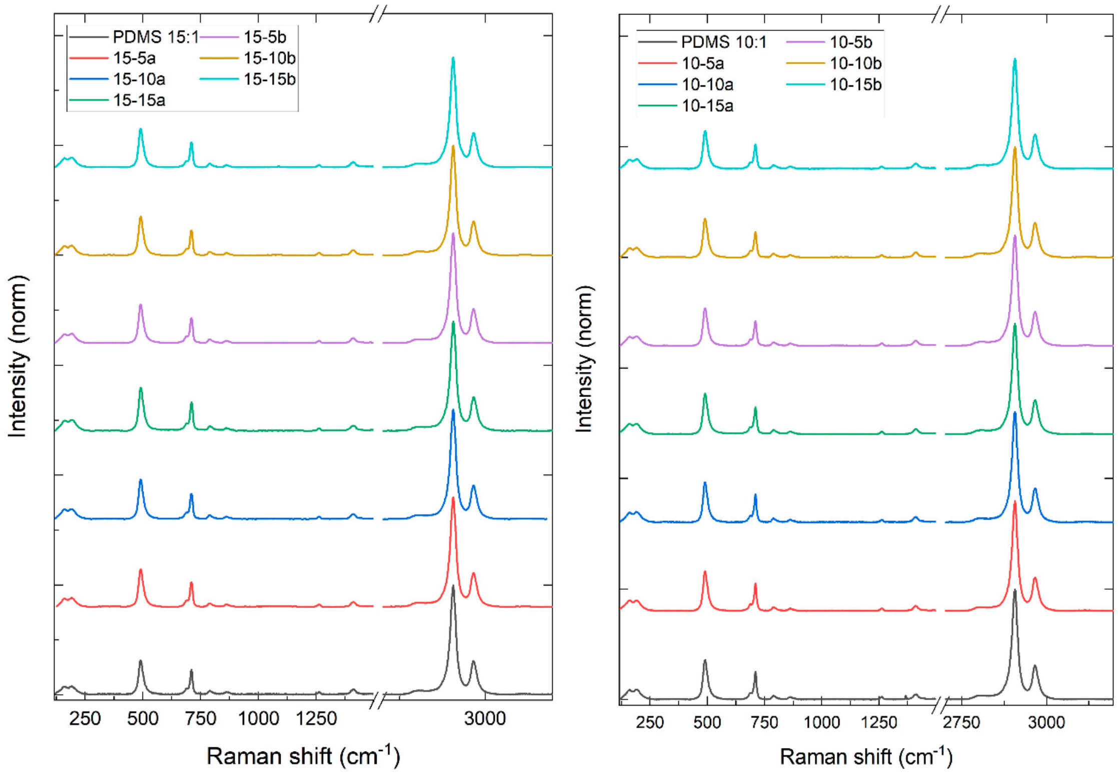

(a) (b)

Figure S2. Raman spectra of (a) PDMS 15:1 and (b) PDMS 10:1 composite with different NPs SiO_2 concentration (5%, 10% and 15%).

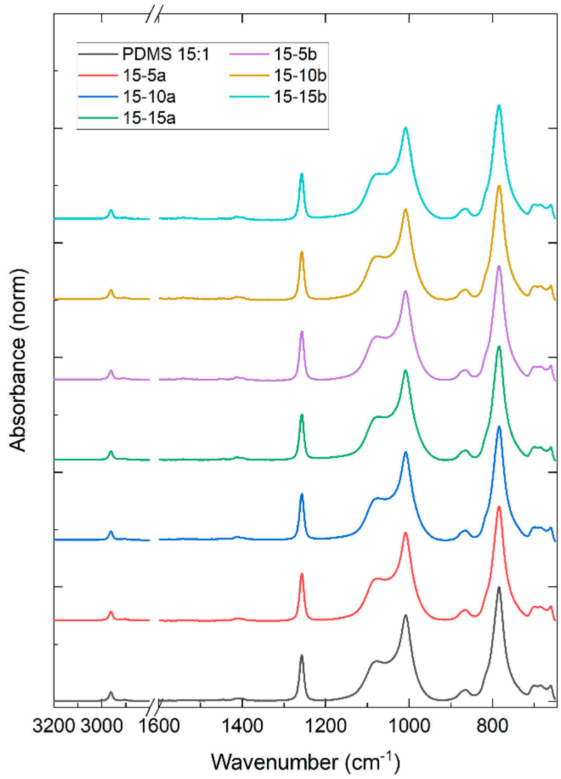

(a)

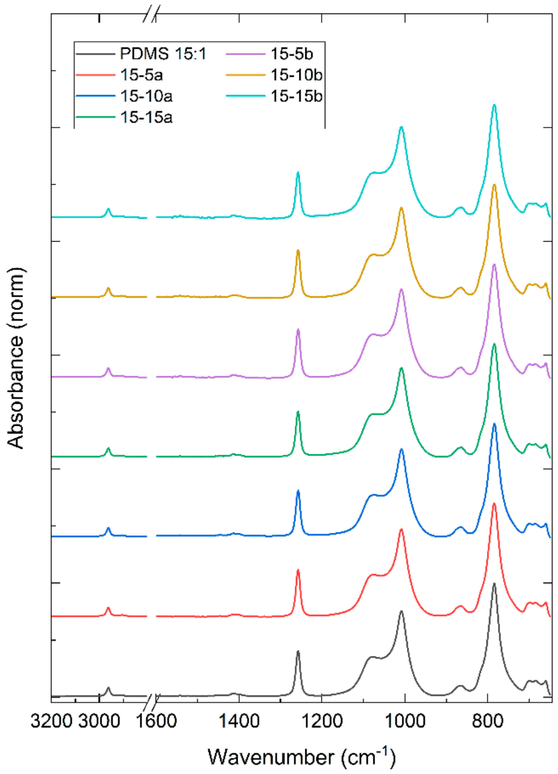

(b)

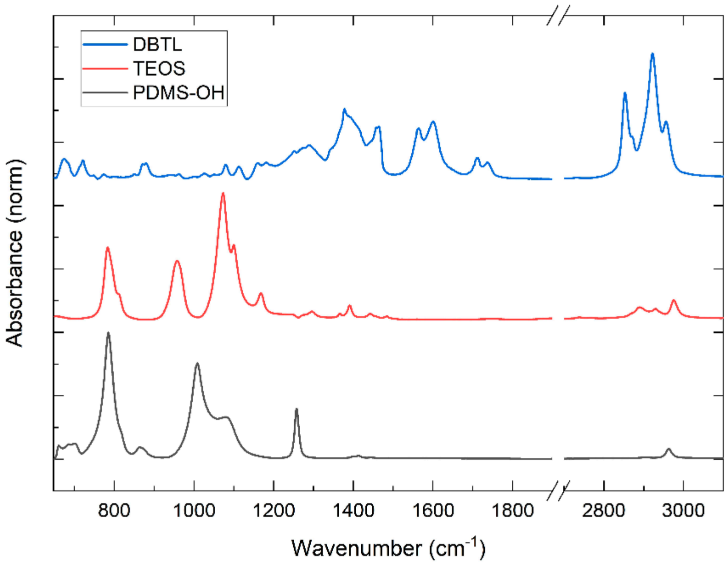

(c)

Figure S3. FTIR spectra of (a) PDMS 15:1 and (b) PDMS 10:1 composite with different NPs SiO_2 concentration (5%, 10% and 15%). (c) FTIR spectra of principal components of PDMS-SiO_2 composites.

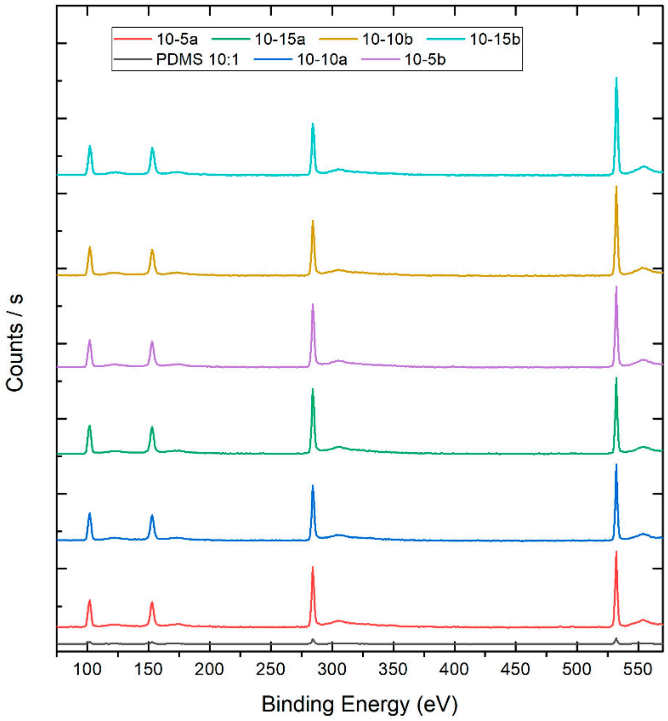

(a)

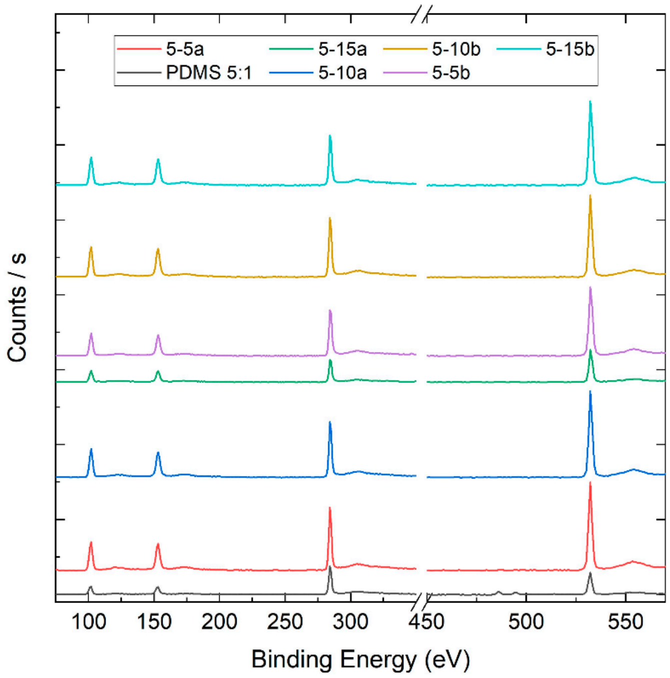

(b)

Figure S4. XPS spectra of (a) PDMS 10:1 and (b) PDMS 5:1 composite with different NPs SiO_2 concentration (5%, 10% and 15%).

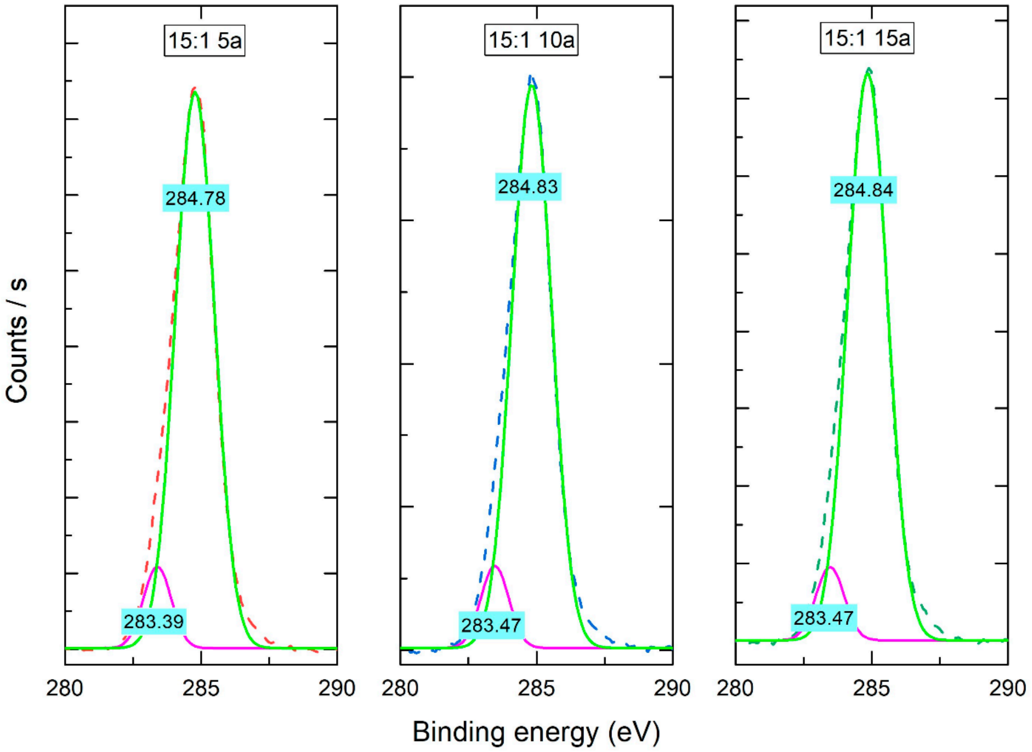
15:1 5a
284.78
283.39
15:1 10a
284.83
283.47
15:1 15a
284.84
283.47
Counts / s
280
285
290
Binding energy (eV)

(a)

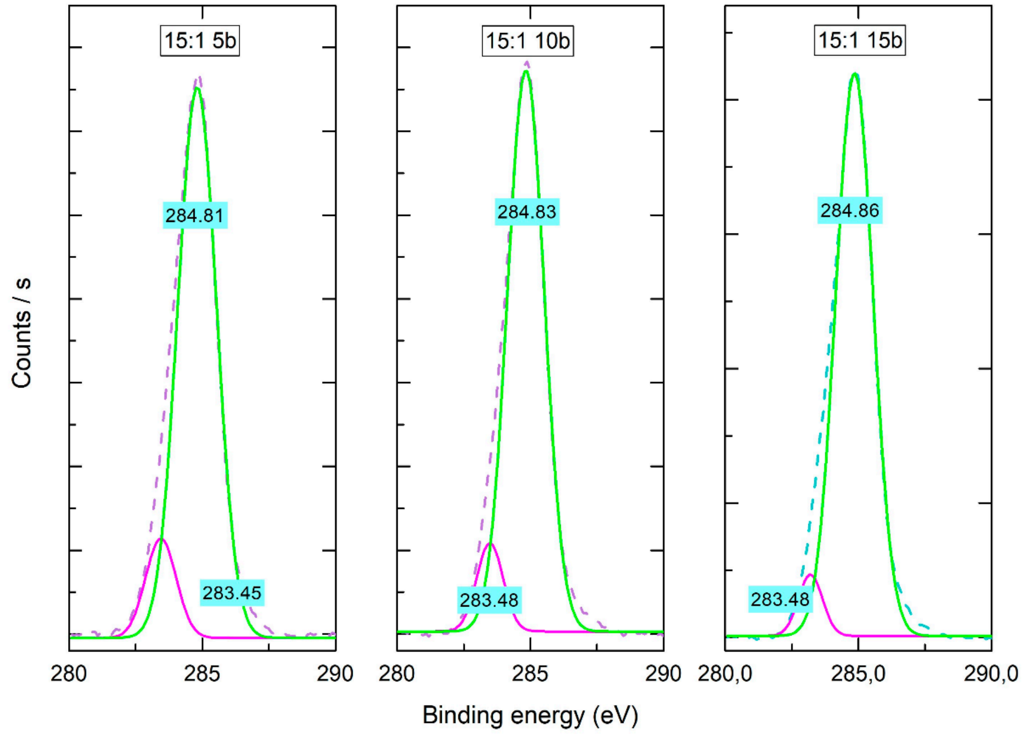
15:1 5b
284.81
283.45
15:1 10b
284.83
283.48
15:1 15b
284.86
283.48
Counts / s
280
285
290
280,0
285,0
290,0
Binding energy (eV)

(b)

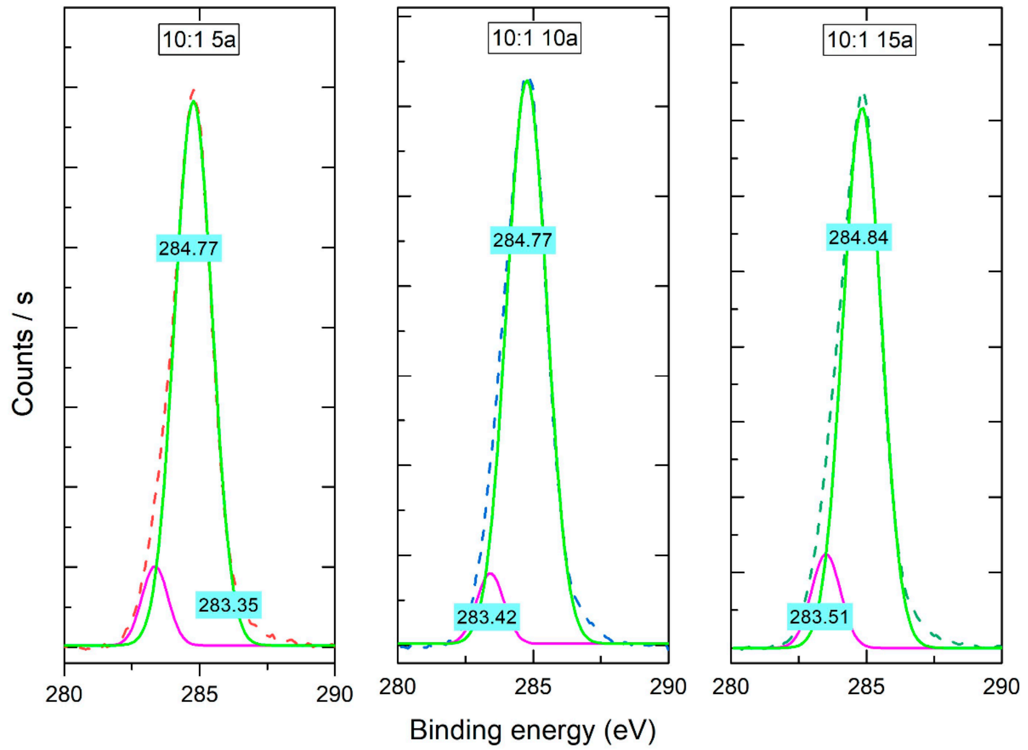
10:1 5a
10:1 10a
10:1 15a
284.77
284.77
284.84
283.35
283.42
283.51
Counts / s
280
285
290
Binding energy (eV)

(c)

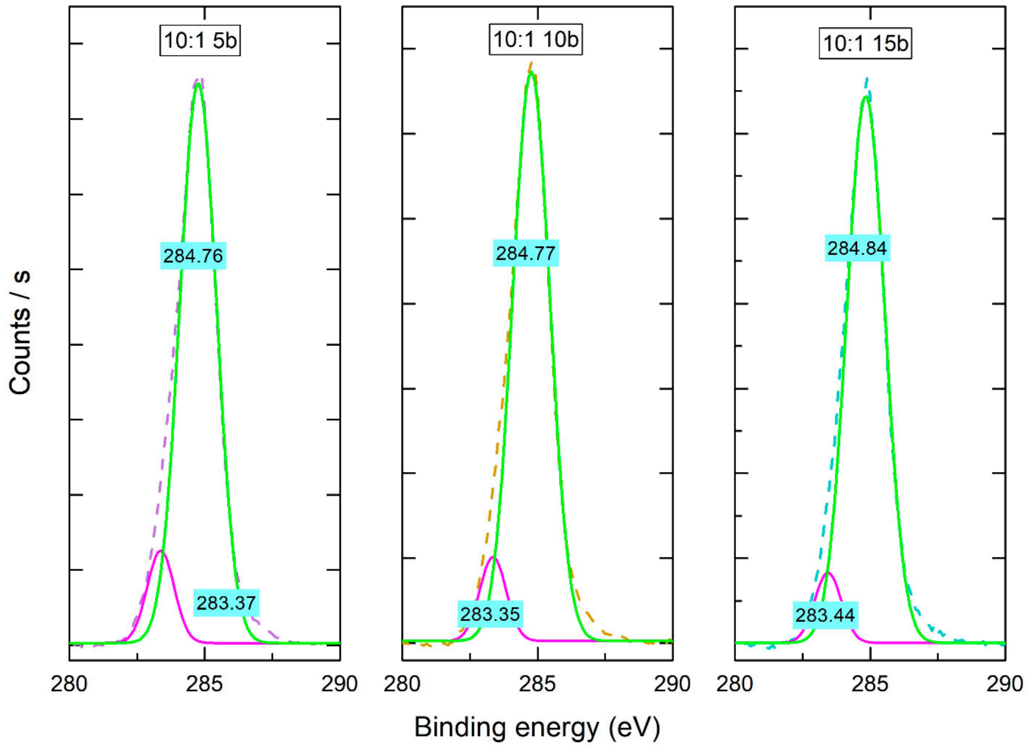
10:1 5b
10:1 10b
10:1 15b
284.76
284.77
284.84
283.37
283.35
283.44
Counts / s
280
285
290
Binding energy (eV)

(d)

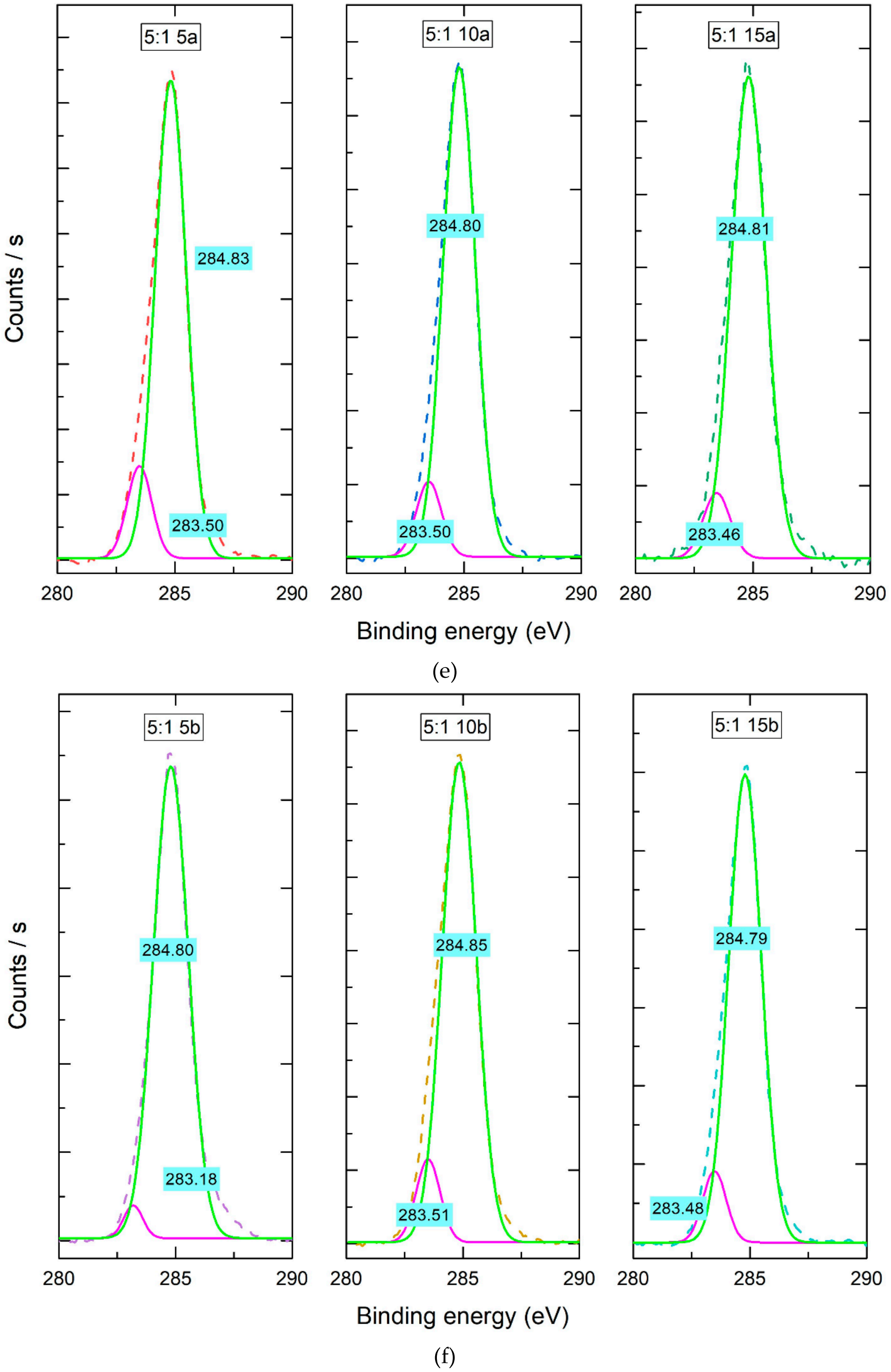

Figure S5. Deconvolution analysis for C1s species of PDMS-%SiO_2 composites: C1s (dash), C-Si (magenta) and C-C (green).

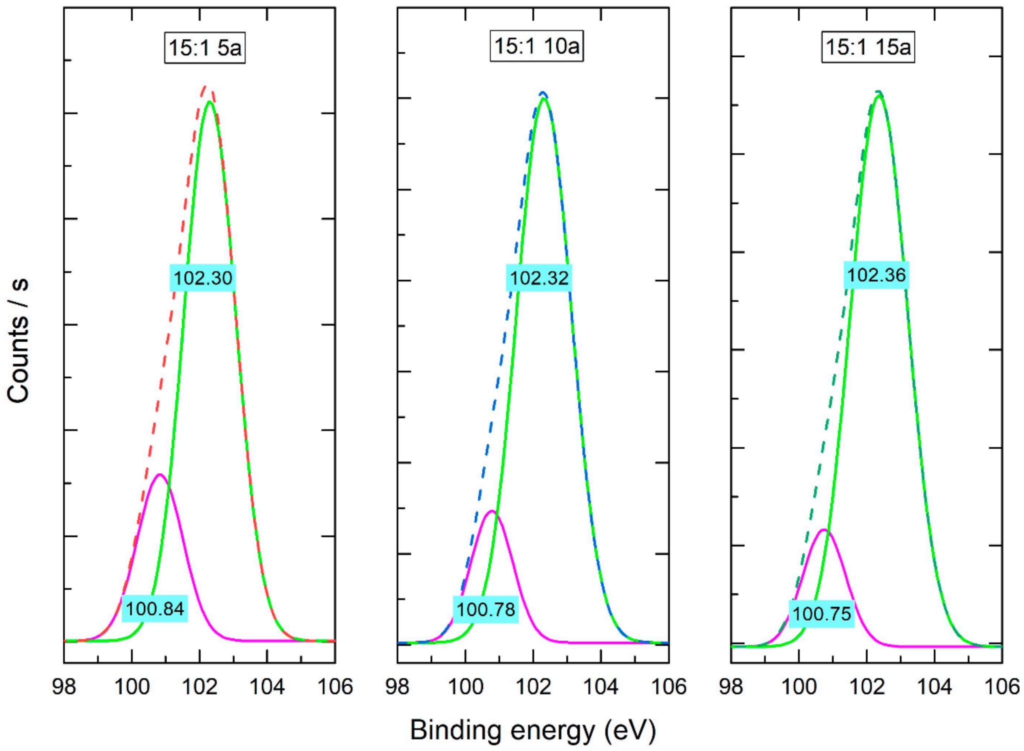
15:1 5a
15:1 10a
15:1 15a
102.30
100.84
102.32
100.78
102.36
100.75
Counts / s
98
100
102
104
106
Binding energy (eV)

(a)

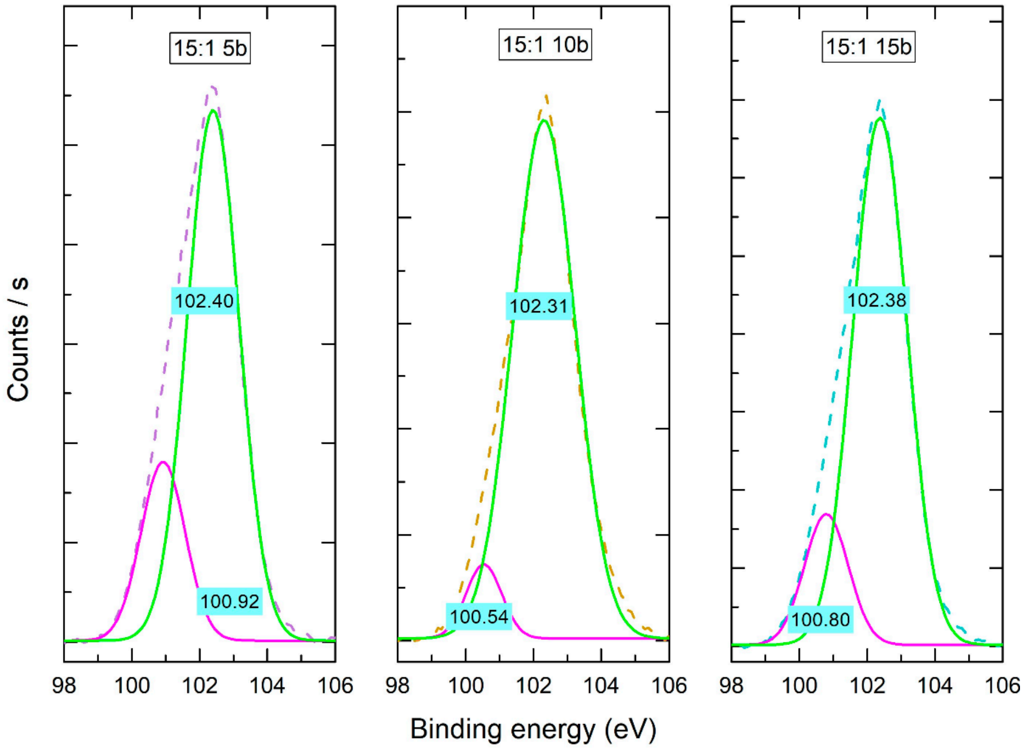
15:1 5b
15:1 10b
15:1 15b
102.40
100.92
102.31
100.54
102.38
100.80
Counts / s
98
100
102
104
106
Binding energy (eV)

(b)

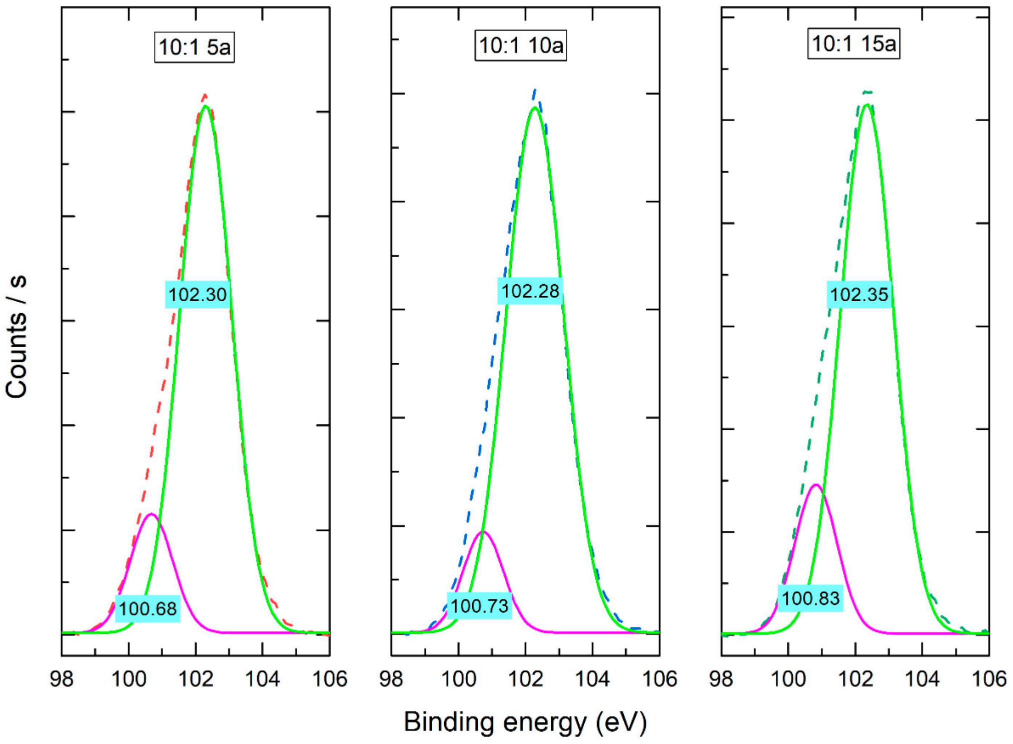
10:1 5a
10:1 10a
10:1 15a
102.30
100.68
102.28
100.73
102.35
100.83
Counts / s
98
100
102
104
106
Binding energy (eV)
(c)

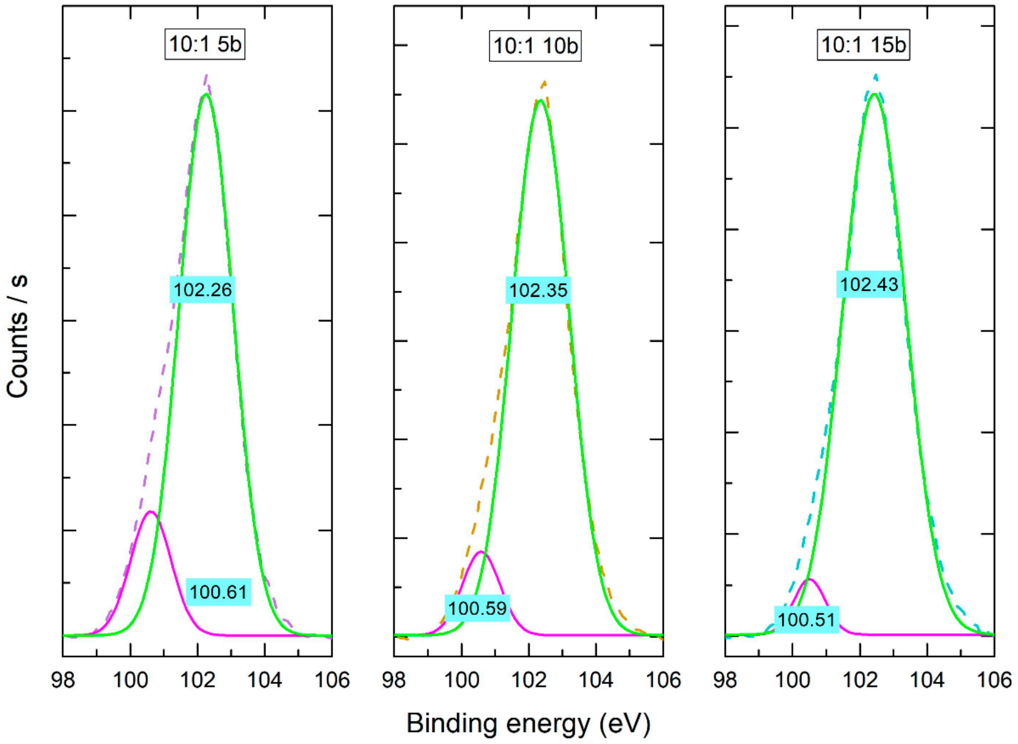
10:1 5b
10:1 10b
10:1 15b
102.26
100.61
102.35
100.59
102.43
100.51
Counts / s
98
100
102
104
106
Binding energy (eV)
(d)

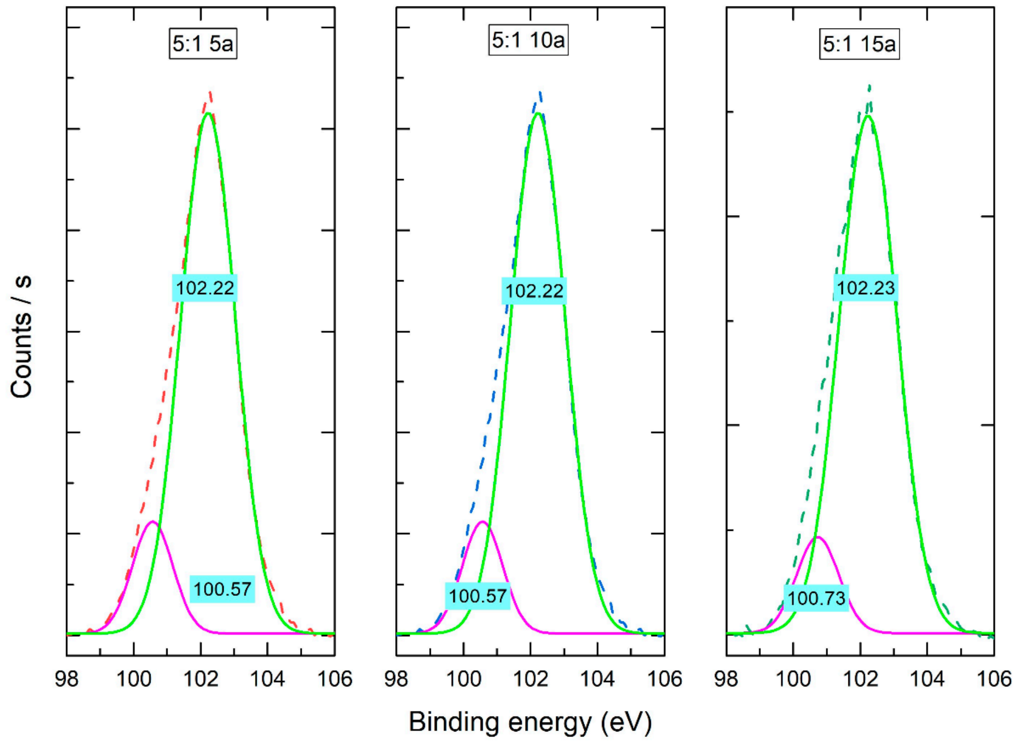

(e)

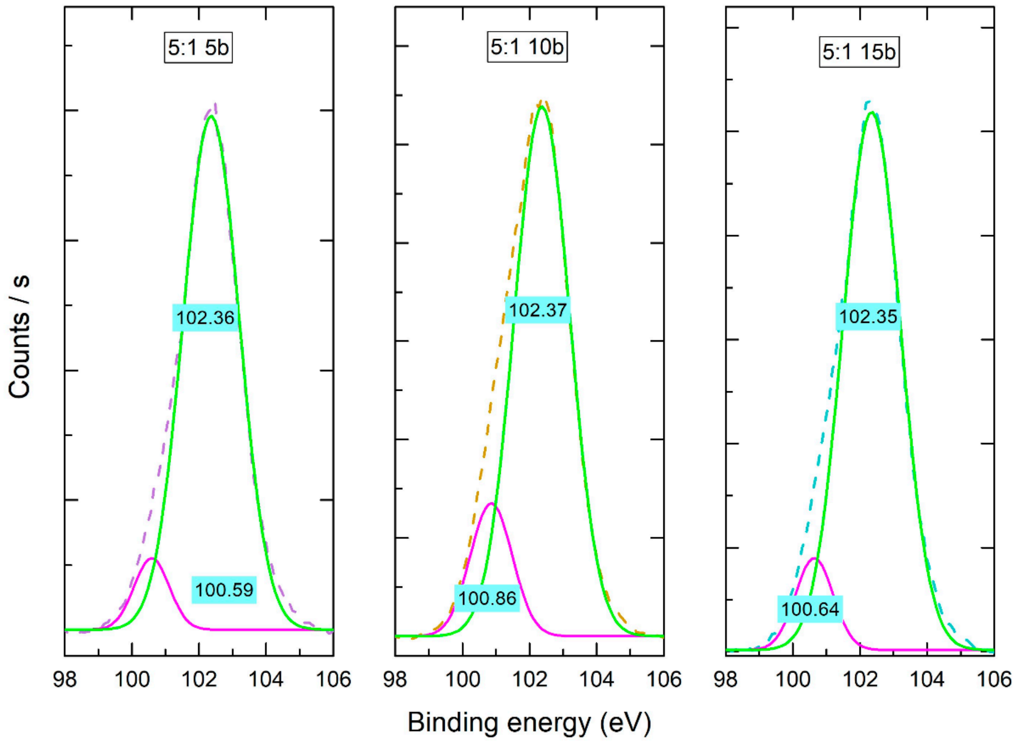

(f)

Figure S6. Deconvolution analysis for Si2p species of PDMS-%SiO_2 composites: Si2p (dash), Si-C (magenta) and O-Si-O (green).
